# Carboxyl truncation of α-synuclein occurs early and is influenced by human APOE genotype in transgenic mouse models of α-synuclein pathogenesis

**DOI:** 10.1186/s40478-023-01623-9

**Published:** 2023-07-23

**Authors:** Grace M. Lloyd, Brooke Long, Stephan Quintin, Zachary A. Sorrentino, Kimberly-Marie M. Gorion, Brach M. Bell, Denise Carrillo, Patrick Sullivan, David Borchelt, Benoit I. Giasson

**Affiliations:** 1 Department of Neuroscience, College of Medicine, University of Florida, BMS J483/CTRND, 1275 Center Drive, Gainesville, FL 32610 USA; 2 Center for Translational Research in Neurodegenerative Disease, College of Medicine, University of Florida, Gainesville, FL 32610 USA; 3 McKnight Brain Institute, College of Medicine, University of Florida, Gainesville, FL 32610 USA; 4 Department of Medicine, Duke University School of Medicine, Durham, NC USA

**Keywords:** Alpha-synuclein, Post-translational modifications, Synucleinopathies, Parkinson’s disease, Dementia with Lewy bodies, Multiple system atrophy, Alzheimer’s disease with amygdala predominant Lewy bodies, C-terminal truncation, APOE

## Abstract

Post-translational modifications to the carboxyl (C) terminus domain of α-synuclein can play an important role in promoting the pathologic aggregation of α-synuclein. Various cleavages that diminish this highly charged, proline-rich region can result in exposure of hydrophobic, aggregation-prone regions, thereby accelerating the aggregation kinetics of α-synuclein into misfolded, pathologic forms. C-terminally truncated forms of α-synuclein are abundant in human diseased brains compared to controls, suggesting a role in disease pathogenesis. Factors that alter the homeostatic proteolytic processing of α-synuclein may ultimately tip the balance towards a progressive disease state. Apolipoprotein E (APOE) has been implicated in the acceleration of cognitive impairment in patients with Lewy body diseases. The APOE4 isoform has been found to cause dysregulation in the endosomal–lysosomal pathway, which could result in altered α-synuclein degradation as a potential mechanism for promoting its pathologic misfolding. Herein, we investigate the spatiotemporal accumulation of C-terminally truncated α-synuclein in a seeded and progressive mouse model of synucleinopathy. Furthermore, we study how this process is influenced in the context of mice that are altered to express either the human APOE3 or APOE4 isoforms. We found that specific C-terminal truncation of α-synuclein occurs at early stages of pathogenesis. We also found that proteolytic processing of this domain differs across various brain regions and is influenced by the presence of different human APOE isoforms. Our data demonstrate an early pathogenic role for C-terminally truncated α-synuclein, and highlight the influence of APOE isoforms in modulating its impact.

**Supplementary Information:**

The online version contains supplementary material available at 10.1186/s40478-023-01623-9.

## Introduction

Parkinson’s disease (PD) is a devastating neurodegenerative disorder, characterized by the slow, progressive loss of motor movement function and control due to degeneration, and eventual death, of dopaminergic neurons in the substantia nigra pars compacta [35]. Molecular investigations into the pathophysiology of PD has revealed the misfolding, aggregation and progressive accumulation of α-synuclein (αSyn) as inclusion pathology, termed Lewy bodies (LBs), to be a major recurrent feature in this disease [5]. Furthermore, this protein has been found to accumulate in many other neurodegenerative diseases, termed synucleinopathies [16]. Common symptoms of synucleinopathies include progressive motor dysfunction, however, cognitive features can be a major component, especially for dementia with Lewy bodies [31, 36]. Recent investigations into synucleinopathies with cognitive features have uncovered curious connections to a major Alzheimer’s disease (AD)—associated gene, apolipoprotein E (*APOE;* HGNC:613*)*. Genome-wide association studies (GWAS) and meta-analyses have exposed the ε4 allele variant of the *APOE* gene (*APOE* ε4) as a contributing risk factor for increased rate of cognitive decline, worsened cognitive impairment and/or earlier onset of dementia in synucleinopathies with cognitive features [4, 9, 18, 21, 30, 34, 37–39, 41, 48, 49]. Studies on the role of *APOE* genotype and cognition in PD provide evidence that *APOE* may directly impact the pathological progression of αSyn in PD [9, 11, 22, 48, 49]; subsequently, in vivo investigations have demonstrated an *APOE* ε4 mediated exacerbation of αSyn pathology in murine models of synucleinopathy [9, 52]. Furthermore, protein levels of APOE dramatically increase in humans with PD [51], as well as in diseased, transgenic αSyn models with PD-related mutations [14]. These findings confirm a modulatory role for APOE in the pathogenesis of αSyn, however, the molecular mechanisms underlying these observations are not well understood.

One possible mechanism by which APOE influences αSyn pathogenesis is via the dysregulation of autophagic processes in an isoform-dependent manner. Aberrant proteolysis is a major feature in synucleinopathies and is linked to increased αSyn accumulation [3, 6, 12, 15, 27, 32]. αSyn proteolysis leads to the formation of cytotoxic, partially degraded, αSyn fragments [17], and, importantly, carboxy-terminally truncated forms of αSyn (αSynΔC), which are hypothesized to be a key precipitating component of αSyn pathogenesis [2, 25, 28, 44]. In the studies herein, we explored the supposition that αSynΔC contributes to αSyn pathogenesis, as well as the effect of *APOE* genotype on this process. Using antibodies specific for αSynΔC-103, -114, -122, -125 and -129, which we have recently established, we assessed the extent to which intramuscular (IM), peripheral seeding with αSyn pre-formed fibrils (PFFs) injection into M83 αSyn transgenic animals resulted in the temporal and spatial pathological formation and distribution of αSynΔC species. Furthermore, using M83 transgenic animals altered on either a human APOE ε3^+/+^ or and human APOE ε4^+/+^ background, we investigated the effect of *APOE* genotype on modulating the accumulation of these proteolytically cleaved species.

## Materials and methods

### Animal research ethics statement

All animal experimental procedures were performed in accordance to University of Florida Institutional Animal Care and Use Committee regulatory policies following approval.

### Mouse lines and tissue sources

This study utilized a combination of archival tissues from a previous time course study of Tg(Prnp-SNCA*A53T)^+/−^, abbreviated hereinafter as TgM83^+/−^ [45], as well as newly generated animals. For the time course study, tissues from a total of 38 TgM83^+/−^ animals were included in this study, 33 of which had been injected intramuscularly with mouse αSyn preformed fibrils (PFFs) and 5 were naïve animals (Summarized in Table 1).

**Table 1:** Summary of animals used in time-course study

Time-course study	
Animal model	TgM83^+/−^ (C3H/BL6)
Cohorts	1 m.p.i.; n = 8 (4F:4M)
	2 m.p.i.; n = 9 (5F:4M)
	3 m.p.i.; n = 8 (4F:4M)
	Aged until paralysis; n = 8 (5F:3M)
	naïve; n = 5 (4F:1M)
Type/amount of injection	10 µg of mouse αSyn PFFs in 5 µl PBS (20 µg total); or PBS
Age of injection	2 months
Region of injection	Bilateral injection in gastrocnemius muscle
Age at harvest	1 m.p.i., 2 m.p.i., 3 m.p.i. and at onset of motor impairment
Total n	38

M83 mice that co-express humanized APOE alleles were newly generated for this study. APOE targeted replacement mice with either the homozygous APOE ε3 or APOE ε4 humanized alleles [23, 46, 47] were obtained from Dr. Patrick Sullivan (Duke University, Durham, NC). TgM83^+/+^ mice were crossed with APOE ε3^+/+^ or APOE ε4^+/+^ mice to produce the F1 generation; 100% of the offspring were heterozygous for the humanized APOE and TgM83 alleles. Pups from the F1 generation were then backcrossed with the respective homozygous humanized APOE mice, and screened for mice that are homozygous for the APOE ε3 or APOE ε4 alleles and positive for the TgM83 allele resulting in APOE ε3^+/+^/M83^+/−^ or APOE ε4^+/+^/M83^+/−^ mice. Mice were mated with respective APOE ε3^+/+^ or APOE ε4^+/+^ while screening for the TgM83 allele for two additional generation to be used for the studies. Since humanized APOE ε3^+/+^ and APOE ε4^+/+^ mice are on a C57BL/6J background, for comparison, TgM83^+/+^ mice were mated with non-transgenic (nTg) C57BL/6J mice (Jackson Laboratory) to generate TgM83^+/−^ that were subsequently maintained in a C57BL/6 J for 3–4 generations. Mice were housed in a stable environment with a 12-h light/dark cycle and access to food and water ad libitum. 51 mice were used in total including: 8 TgM83^+/−^ mice, all of which were injected with pre-formed fibrils (PFFs) (6F:2M), 24 APOE ε3^+/+^/M83^+/−^ mice, 12 (6F:6M) injected with phosphate buffered saline (PBS), 12 (6F:6M) injected with PFFs, and 19 APOE ε4^+/+^/M83^+/−^ mice, 7 (3F:4M) injected with PBS, 12 (6F:6M) injected with PFFs. Mouse data is summarized in Table 2.

**Table 2:** Summary of animals used in APOE genotype study

APOE genotype study			
Animal model	TgM83^+/−^ (BL6)	APOE ε3^+/+^/TgM83^+/−^	APOE ε4^+/+^/TgM83^+/−^
Cohorts	PFF-injected; n = 8 (6F:2M)	PFF injected; n = 12 (6F:6M)	PFF injected; n = 12 (6F:6M)
		PBS injected; n = 12 (6F:6M)	PBS injected; n = 7 (3F:4M)
Type/amount of injection	5 µg of human αSyn PFFs in 5 µl PBS; or PBS		
Age of injection	2 months		
Region of injection	Unilateral injection in right gastrocnemius muscle		
Age at harvest	8 months post-injection unless motor impaired		
Total n	51		

### αSyn fibril preparation

For the TgM83^+/−^ study with varying APOE isoforms, human PFFs generation was prepared as follows: recombinant human αSyn was expressed in *E. coli* then purified using size exclusion and ion exchange chromatography as previously described [42]. Human αSyn protein (5 mg/ml in sterile phosphate buffered saline (PBS)) was incubated at 37 °C with constant shaking at 1050 RPM (Thermomixer R, Eppendorf) for 5 days to induce fibrillization. Fibril formation was monitored by K114 [(trans, trans)-1-bromo-2,5-bis-(4-hydroxy) styrylbenzene] fluorometry as previously described [7]. Fibrils were diluted to 2 mg/ml in sterile PBS and fragmented via water bath sonication at 40 kHz for 1 h at RT, prior to injection, as previously described [43].

### Gastrocnemius injection and survival analysis

For the time-course study, intramuscular (IM) injection and tissue processing for animals were previously described [45]. Briefly, at 2 months of age, mice were deeply anesthetized with isoflurane (1–5%) inhalation, then bilaterally injected in the gastrocnemius muscles, with 10 µg of mouse αSyn PFFs in 5 µl of sterile PBS. For the APOE/M83^+/−^ study, mice were aged for 2 months, then injected in the right gastrocnemius with 5 µg of human PFFs (1 µg/µl) or sterile PBS. Following inoculation, mice were regularly assessed for motor deficits. For both studies, the survival end point was motor impairment/paralysis, upon which the animals were euthanized. For the APOE/M83^+/−^ study, mice were aged until the onset of motor symptoms or until the pre-determined study end point at 185 days post-injection.

### Development and validation of antibodies specific various forms of αSynΔC

Monoclonal antibodies specific for αSyn carboxy (C)-terminally truncated at residues 103, 114, 122, 125 or 129 [20, 40] (see Table 3) were previously described.

**Table 3:** Summary of antibodies and antigen retrieval methods used in study

Key resources table					
Primary antibodies	Identifier	Specificity	Antigen retrieval for IHC	Source	References
Anti-αSyn pS129, Mouse	81A RRID: AB_2819037	α-syn at pSer129	Water w/0.05% Tween, heat bath	B. Giasson University of Florida College of Medicine; Florida; USA	[50]
p62/ sequestresome-1, Rabbit	p62	Sequestresome1	Water w/0.05% Tween, heat bath	ProteinTech, Rosemont, IL	[24]
Anti-αSyn, Mouse	2H6	αSyn (2–21)	Water w/0.05% Tween, heat bath	B. Giasson University of Florida College of Medicine; Florida; USA	[10]
Anti-αSyn, Mouse	3H19	αSyn (110–119)	DAKO Target Retrieval Solution, heat bath/70% formic acid	B. Giasson University of Florida College of Medicine; Florida; USA	[29]
Anti-αSyn cleaved at 103, Mouse	2G5	αSyn (x-103)	Formalin incubation/DAKO Target Retrieval Solution, heat bath/70% formic acid	B. Giasson University of Florida College of Medicine; Florida; USA	[20]
Anti-αSyn cleaved at 114, Mouse	1A2	αSyn (x-114)	DAKO Target Retrieval Solution, heat bath/70% formic acid	B. Giasson University of Florida College of Medicine; Florida; USA	[40]
Anti-αSyn cleaved at 122, Mouse	10A4	αSyn (x-122)	DAKO Target Retrieval Solution, heat bath/70% formic acid	B. Giasson University of Florida College of Medicine; Florida; USA	[20]
Anti-αSyn cleaved at 125, Mouse	5C1	αSyn (x-125)	DAKO Target Retrieval Solution, heat bath/70% formic acid	B. Giasson University of Florida College of Medicine; Florida; USA	[20]
Anti-αSyn cleaved at 129, Mouse	2G7	αSyn (x-129)	DAKO Target Retrieval Solution, heat bath/70% formic acid	B. Giasson University of Florida College of Medicine; Florida; USA	[20]

### Tissue processing and immunohistochemical analysis

For immunohistochemistry (IHC) studies, mice were euthanized with CO_2_, and perfused with a heparin/PBS solution, as previously described [45]. Briefly, brains and spinal cords of mice were harvested and fixed in 70% EtOH/150 mM NaCl, paraffin embedded, and coronally cut into 5 µm sections, as previously described [45]. Immunostaining was performed using established methods [13]. Briefly, tissue sections were rehydrated with xylenes and graded, 100–70% ethanol steps followed with heat-induced epitope retrieval (HIER) in a steam bath for 60 min using retrieval method indicated in Table 3. After antigen retrieval, sections were washed in running deionized H_2_O for 15 min. For 3H19 antibody and all of the αSyn C-truncation specific antibodies, sections were then treated with 70% formic acid for 10 min, then rinsed in running deionized H_2_O for 15 min. Endogenous peroxidase was quenched by incubating sections in 1.5% hydrogen peroxide/0.005% Triton-X-100 diluted in PBS, pH 7.4 (Invitrogen) for 15–20 min. For antibody 2G5, Triton-X-100 was omitted from this step. Sections were then rinsed in running deionized H_2_O for 15 min. Slides were washed 3 times for 5 min in 0.1 M Tris, pH 7.6, or Tris buffered saline (TBS; 50 mM Tris, pH 7.5, 150 mM NaCl) (for 1A2 only), then blocked for 10 min in 2% fetal bovine serum (FBS)/0.1 M Tris solution or 5%milk/TBS (1A2 only). Sections were incubated with primary antibodies diluted in blocking solution, if required, and stored overnight in 4°. After overnight incubation, primary antibody was removed from slides with a quick rinse, then incubated with agitation for 5 min in 0.1M Tris or TBS (for 1A2 only), 3 times. Tissue sections were incubated for 1 h with biotinylated secondary IgG (Vector Laboratories; Burlingame, CA) in 0.1M Tris, pH 7.6/2% FBS or 5%milk/TBS (for 1A2 only) at 1:3000 at RT. For 3H19 antibody and all of the αSyn C-truncation specific antibodies, ImmPRESS polymer secondary antibody (Vector Laboratories; Burlingame, CA) was diluted in a 1:10 ratio with the standard secondary antibody solution described above. Secondary antibody was rinsed 3 times with 0.1M Tris, or TBS (for 1A2 only), for 5 min each. Sections were then incubated with an avidin–biotin complex (ABC) solution (Vectastain ABC Elite kit; Vector Laboratories, Burlingame, CA) for 1 h at RT, then rinsed again, 3 times, with 0.1M Tris, or TBS (for 1A2 only), for 5 min each. Sections were developed using chromogen 3,3′-diaminobenzidine (DAB kit; KPL, Gaithersburg, MD), rinsed for 15 min in running tap water, then counterstained using hematoxylin (Sigma Aldrich, St. Louis, MO). Summary of antibodies used and corresponding retrieval methods are summarized in Table 3.

### Semi-quantification and digital analysis of pathology

All IHC sections were digitally scanned using an Aperio ScanScope CS instrument (40 × magnification; Aperio Technologies Inc., Vista, CA, USA), and images of representative areas of pathology were captured using the ImageScope software (40 × magnification; Aperio Technologies Inc. Vista, CA, USA). Tissue sections were manually scored for αSyn pathology on a scale of 0 (no pathology) to 3 (highest pathology) by three independent raters using the Allen Brain Atlas to define regions (Allen Reference Atlas—Mouse Brain [brain atlas]. Available from atlas.brain-map.org) [1, 8, 19, 26, 33]. Morphological descriptions were classified using categories defined previously [29]. Representative images were corrected for color/hue values; brightness/contrast adjustments were applied identically on captured images within each figure using Adobe Photoshop CS3 (Adobe Systems, San Jose, CA, USA). All raw files are available upon request*.*

### Statistical analysis

Experiments were analyzed using parametric statistical tests described in detail in the figure legends. No animals were excluded from analysis. Data was tested for normality using D’Agostino-Pearson omnibus test. Statistical analysis was performed using Prism software (GraphPad Software, San Diego, CA, USA) and detailed in figure legends. For time-course experiments, results were analyzed using 2-way ANOVA and corrected for multiple comparisons using the Tukey test. Kaplan–Meier curves of motor phenotype development were compared using Log-rank (Mantel–Cox). Column analysis of data with more than 2 groups were analyzed by 1-way ANOVA corrected for multiple comparisons using Tukey’s test. For 2-way ANOVAs, simple effects comparing the mean between groups was reported (detailed in figures). Graphs depicting correlations were computed using Pearson’s correlation coefficients (Pearson r and two-tailed *p*-value annotated on graphs) and shown with a simple linear regression line with 95% confidence bands of the best-fit line. *P* values were multiplicity adjusted, family-wise significance was set at 0.05 and data are presented as mean ^+/−^ SEM.

## Results

### Immunohistopathological analysis of PFF intramuscularly injected TgM83^+/−^ mice using novel αSynΔC specific antibodies

**Fig. 1:**
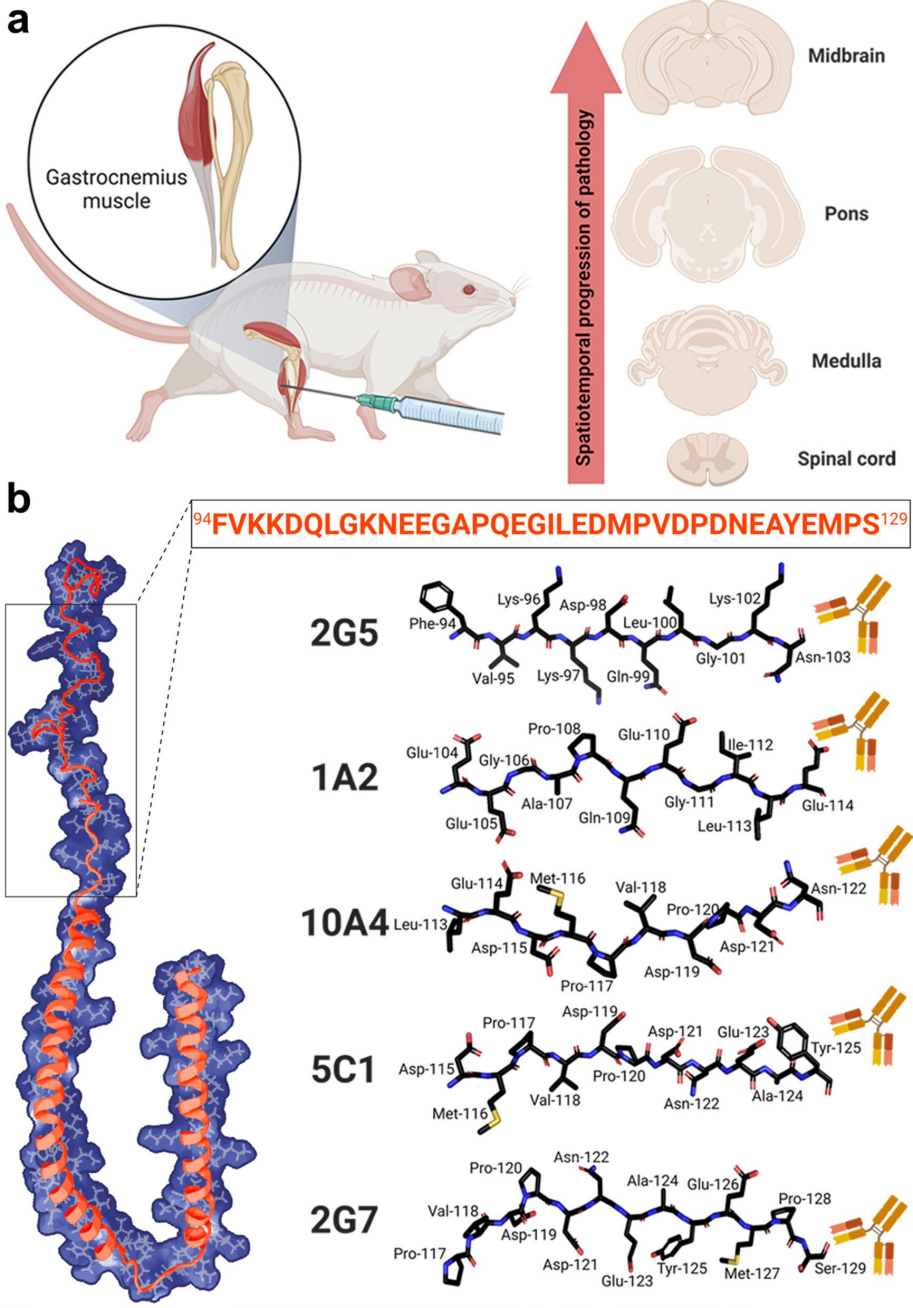
Schematics of experimental designs assessing the spatiotemporal progression of αSyn inclusion pathology including various αSynΔC forms. (**a**) Representative illustration of experimental design showing IM injection of PFFs and theorized route of spatiotemporal pathological propagation of αSyn type prion transmission. (**b**) Stick diagrams of peptide sequences used to generate αSynΔC specific αSyn antibodies. Antibodies are not drawn to scale. Structural model is based on PDB accession 1XQ8. Created with Biorender.com

To assess the spatiotemporal progression of αSynΔC pathology, we analyzed archival tissues from a cohort of animals that have previously published [45]. In that study, TgM83^+/−^ mice were injected bilaterally in the gastrocnemius muscle with mouse PFFs, or PBS at 2 months of age. PFF-injected mice were harvested at 1-, 2-, or 3- months post injection (m.p.i.), or upon paralysis; naïve mice control mice were harvested at the same timepoints (Fig. 1a).

We previously reported that spinal motor neuron degeneration in TgM83^+/−^ mice, begins at approximately 2 months post-IM injection even though pSer129 positive αSyn pathology is limited [45]. We also found that pSer129 positivity increased over time and throughout neuroanatomically connected regions, in PFF-injected mice, while naïve mice display no αSyn pathology [45]. In this study, IHC analysis was performed with αSynΔC specific antibodies, generated and characterized previously, [20, 40] targeting αSynΔC truncated at residues 103, 114, 122, 125, and 129 (Fig. 1b). We investigated whether αSynΔC positivity is detected in regions distal to injection site, and whether burden of αSynΔC positive inclusions increase with age. Similar to staining for pSer129, αSynΔC positivity was not detected in naïve mice [45] (data not shown).

### αSynΔC forms of αSyn are detected in the spinal cord at 2-months post IM injection of PFFs and increase temporally

**Fig. 2:**
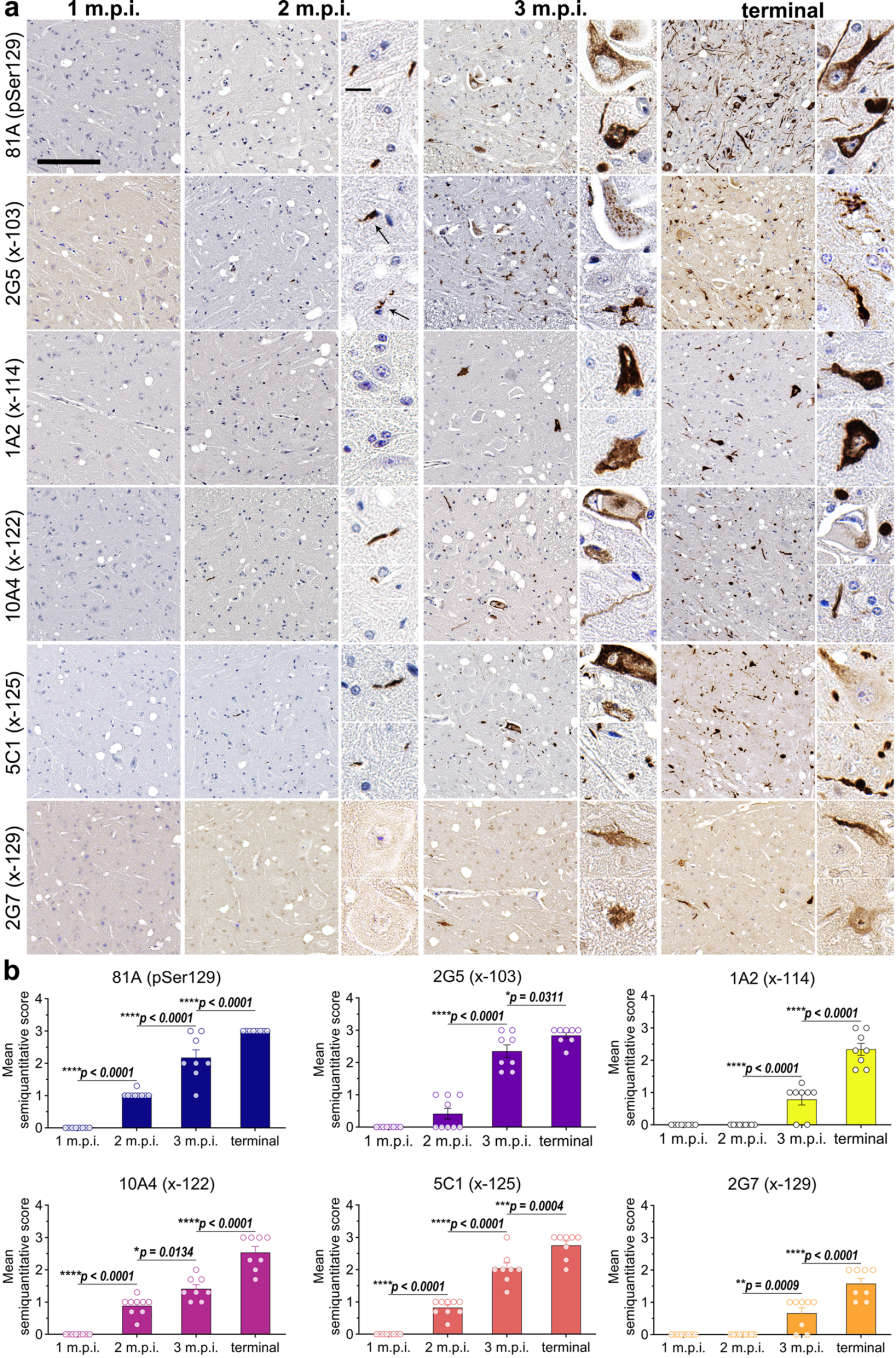
Progressive accumulation of αSyn pathology and αSynΔC forms in the spine of TgM83^+/−^ mice following peripheral intramuscular administration of PFFs. (**a**) Representative IHC images comparing αSyn pathological deposition in TgM83^+/−^ mice at 1-, 2-, or 3- months post IM injection or terminal stage. Antibodies specific for αSyn phosphorylated at Ser129 (81A) and αSynΔC at residues 103 (2G5), 114 (1A2), 122 (10A4), 125 (5C1) and 129 (2G7) were used for IHC, as indicated. Sections were counterstained with hematoxylin. Scale bar for high magnification images = 150 μm; for inset = 15 μm. (**b**) Semi-quantification of antibody immunoreactivity for αSyn inclusion pathology. Data expressed as mean +/− SEM. Results were analyzed using 2-way ANOVA and corrected for multiple comparisons using Tukey’s test. n = 8 (4F:4M); 9 (5F:4M); 8 (4F:4M); 8 (5F:3M). *p < 0.05; **p < 0.01; ***p < 0.001; ****p < 0.0001

Following IM injection of PFFs in TgM83^+/−^ mice, αSyn inclusion pathology was not detected at 1 m.p.i. as it is first detected at 2 m.p.i. in the intermediate and ventral regions of the spinal cord (Fig. 2) [45]. Therefore, to investigate the earliest occurrence of αSynΔC pathology, we probed spinal tissue with antibodies targeting pSer129 and αSynΔC-103, -114, -122, -125 and -129. Three different C-terminally truncated forms of αSyn, αSynΔC-103 (2G5), αSynΔC-122 (10A4), and αSynΔC-125 (5C1), were detectable in inclusions by 2 m.p.i. (Fig. 2). 2G5 (αSynΔC-103) positive inclusions were found in the anterior horn and intermediate region (Additional file 1: Fig. S1), predominantly as smaller, perinuclear aggregates in glial cells and punctate processes in the neuropil (Fig. 2a). 10A4 (αSynΔC-122) positive inclusions were found in the anterior horn, 5C1 (αSynΔC-125) positive inclusions were found in the lateral anterior horn and intermediate region, and 81A (pSer129) positive inclusions were detected in the anterior and intermediate regions (Additional file 1: Fig. S1) **(**Fig. 2a). 10A4, 5C1 and 81A positive inclusions shared morphological characteristics, appearing as neuritic processes within the neuropil. By 3 m.p.i., all αSynΔC-specific antibodies detected positive inclusions in the spine, and by terminal endpoint, positive αSyn pathology was detected in both dorsal and ventral regions by all antibodies (Additional file 1: Fig. S1), and these inclusions were larger in size (Fig. 2a). Temporal analysis of 81A and 5C1 positive pathology revealed a linear increase between 2 m.p.i. and terminal endpoint (Fig. 2b). Comparatively, 2G5 pathology was relatively low at 2 m.p.i., but increased greatly in abundance by 3 m.p.i. (Fig. 2b). 1A2 and 2G7 reactive pathology was moderate at 3 m.p.i. and approximately doubled between 3 m.p.i. and end stage (Fig. 2b). 10A4 pathology was moderate at 2 m.p.i., displayed a modest increase at 3 m.p.i., and nearly doubled by terminal endpoint (Fig. 2b). Overall, by terminal endpoint, 2G7 (x-129) immunoreactivity revealed the lowest burden of inclusions, while 2G5 (x-103) staining had the highest (Fig. 2b).

### αSynΔC-103 and αSynΔC-125 positive pathology detected at 2 m.p.i. in hindbrain

**Fig. 3:**
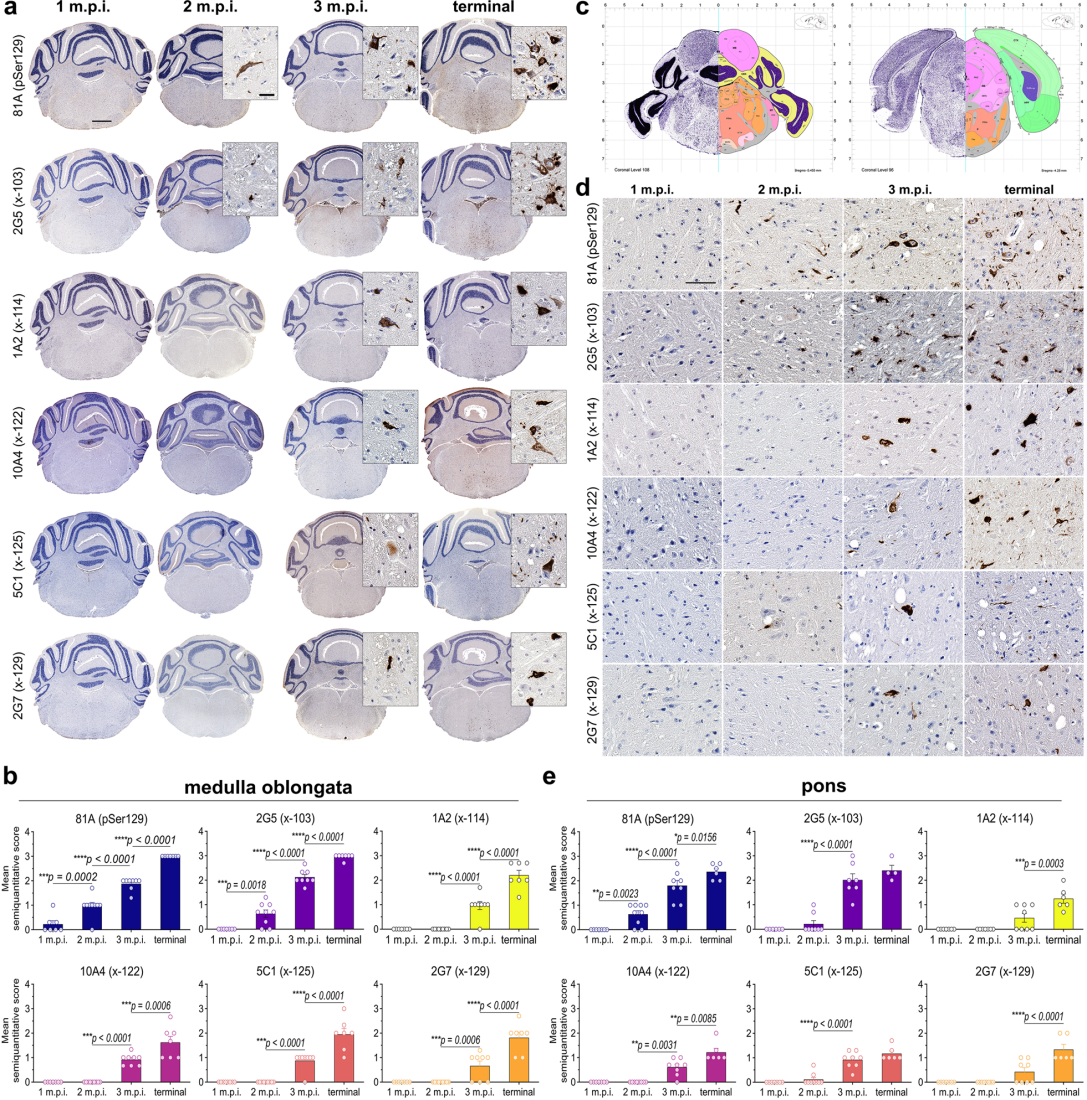
Detection of αSynΔC truncated at residues 103 and 125 in the hindbrain of TgM83^+/−^ mice 2 months after peripheral intramuscular PFF inoculation. Representative IHC images and semiquantitative analysis comparing pathological αSyn deposition in TgM83^+/−^ mice at 1-, 2-, 3- months post IM injection or terminal stage in (**a**, **b**) the medulla and (**c**–**e**) the pons. Antibodies specific for αSyn phosphorylated at Ser129 (81A) and αSynΔC truncated at residues 103 (2G5), 114 (1A2), 122 (10A4), 125 (5C1) and 129 (2G7) were used for IHC, as indicated. Sections were counterstained with hematoxylin. **c** Nissl (left) and anatomical annotations (right) of coronal section panels from the Allen reference atlas of the adult mouse brain, pons regions analyzed in study are annotated in salmon. Scale bar = 1 mm for low magnitude image and 30 μm for inset in (**a**) and 60 μm in (**d**). Data represents mean +/− SEM. Results were analyzed using 2-way ANOVA and corrected for multiple comparisons using Tukey’s test. n = 8 (4F:4M); 9 (5F:4M); 8 (4F:4M); 8 (5F:3M). *p < 0.05; **p < 0.01; ***p < 0.001; ****p < 0.0001

In order to determine whether the temporal pattern of αSynΔC-positive inclusions continues in regions increasingly distal to the injection site, pathology in the hindbrain regions were assessed. In the medulla, the only form of C-terminally truncated αSyn detected at 2 m.p.i. was αSynΔC-103 with antibody 2G5, however, all of αSyn C-truncation epitopes were detected at 3 m.p.i. (Fig. 3a–b). By the terminal endpoint, 2G5 immunoreactive pathology was extensive and widespread in the medulla, including in the reticular formation and lateral vestibular nuclei (LAV) (Fig. 3a). 2G5 inclusions were of distinct morphology, appearing as perinuclear and intranuclear dots, web-like, and ringed cell body inclusions, lightning, corkscrew, and beaded neurites, axonal spheroids and dot-like neuropil (Fig. 3a). Of note, 2G5 inclusion pathology was rare or not found in the interpolar part of the spinal nucleus of the trigeminal (SPVI), inferior olivary complex (IO), or nucleus prepositus (PRP). By the terminal endpoint, medullary 1A2 immunoreactive pathology was regionally constrained, primarily to the gigantocellular reticular nucleus (GRN), the lateral paragigantocellular reticular nucleus (PGRNI), the ventral medullary reticular nucleus (MDRNv), and the magnocellular reticular nucleus (MARN), and rarely in the LAV. (Fig. 3a). By terminal endpoint, medullary 10A4 positive pathology was restricted to the MDRNv, GRN, MARN, PGRNI, the hypoglossal nucleus (cranial nerve (CN) XII), the magnocellular part of the lateral reticular nucleus (LRNm), oral part of the spinal nucleus of the trigeminal (SPVO), spinal vestibular nucleus (SPIV), intermediate reticular nucleus (IRN) and rarely in the LAV, as distorted nucleus, LB-like, flame-shaped inclusions, beaded and corkscrew neurites, dot-like neuropil and neuropil threads (Fig. 3a). By terminal endpoint, medullary 5C1 positive pathology was typically detected in the IRN, MDRNv, nucleus of the solitary tract (NTS), superior vestibular nucleus (SUV), CN XII, dorsal motor nucleus of the vagus nerve (DMX), LRNm, PRP, medial vestibular nucleus (MV), SPIV, SPVI, GRN, IRN, PGRNI and LAV as ringed, LB-like, flame-shaped inclusions, beaded, swollen and corkscrew neurites, dot-like neuropil, and neuropil threads (Fig. 3a). By terminal endpoint, medullary 2G7 positive pathology was detected in the GRN, PGRNI, IRN, MDRNv, LAV and LRNm, as distorted nucleus, swollen neurites, flame-shaped, LB-like and ringed inclusions (Fig. 3a). The pattern of increase in pathological positivity over time in the medullary region was different from that observed in the spinal cord. While the spinal cord showed a more sigmoidal increase in pathological positivity over time, the medullary region displayed a gradual, steady escalation in all forms of αSyn positive pathology at each time point (Fig. 3b). This suggests that the accumulation of pathological positivity in the medulla is more consistent over time compared to the spinal cord, which shows a more variable pattern of accumulation.

To analyze regions within the pons for C-terminally truncated forms of αSyn, we immunostained regions illustrated in the annotated reference in Fig. 3c. Along with pSer129 positive pathology, 2G5 and 5C1 were also detected at 2 m.p.i. (Fig. 3d–e). Similar to the pattern revealed in the medulla, all forms of probed C-terminally truncated αSyn were detected at 3 m.p.i. in the pons (Fig. 3d–e). By the terminal endpoint, pontine 2G5 reactive pathology was widespread and appeared as perinuclear dots, LB-like inclusions, axonal spheroids, neuropil threads and dot-like neuropil (Fig. 3d). By the terminal endpoint, pontine 1A2 pathology was moderate and restricted to the pontine reticular nucleus (PRN). 1A2 positive morphology consisted of flame-shaped, globose, dot-like neuropil, neuropil threads, swollen and corkscrew neurites (Fig. 3d). By the terminal endpoint, pontine 10A4 pathology was moderate and limited to the PRN and the nucleus of the lateral lemniscus (NLL). 10A4 positive morphology consisted of distorted nucleus, LB-like, flame-shaped, ringed, and globose inclusions, dot-like neuropil and neuropil threads (Fig. 3d). By the terminal endpoint, pontine 5C1 pathology was moderate and widespread throughout the pons. 5C1 positive inclusions presented as flame-shaped, swollen and corkscrew neurites, dot-like neuropil and neuropil threads (Fig. 3d). By the terminal endpoint, pontine 2G7 pathology was moderate and restricted to the PRN as distorted nucleus, swollen neurites, flame-shaped, globose and ringed inclusions (Fig. 3d). In this region, the rate of pSer129 positive pathology increased steadily across each timepoint, a trend that was roughly recapitulated by 1A2 (αSynΔC-114), 10A4 (αSynΔC-122) and 2G7 (αSynΔC-129) positive pathology (Fig. 3e). In contrast, 2G5 (αSynΔC-103) and 5C1 (αSynΔC-125) positive pathology revealed a more sigmoidal pattern of accumulation over time, wherein a significant spike in immunostaining occurred between 2 and 3 m.p.i., and no significant change in pathology was detected by end stage (Fig. 3e).

### αSynΔC positive inclusions detected in neuroanatomically connected regions distal to injection site by end stage in αSyn mice

**Fig. 4:**
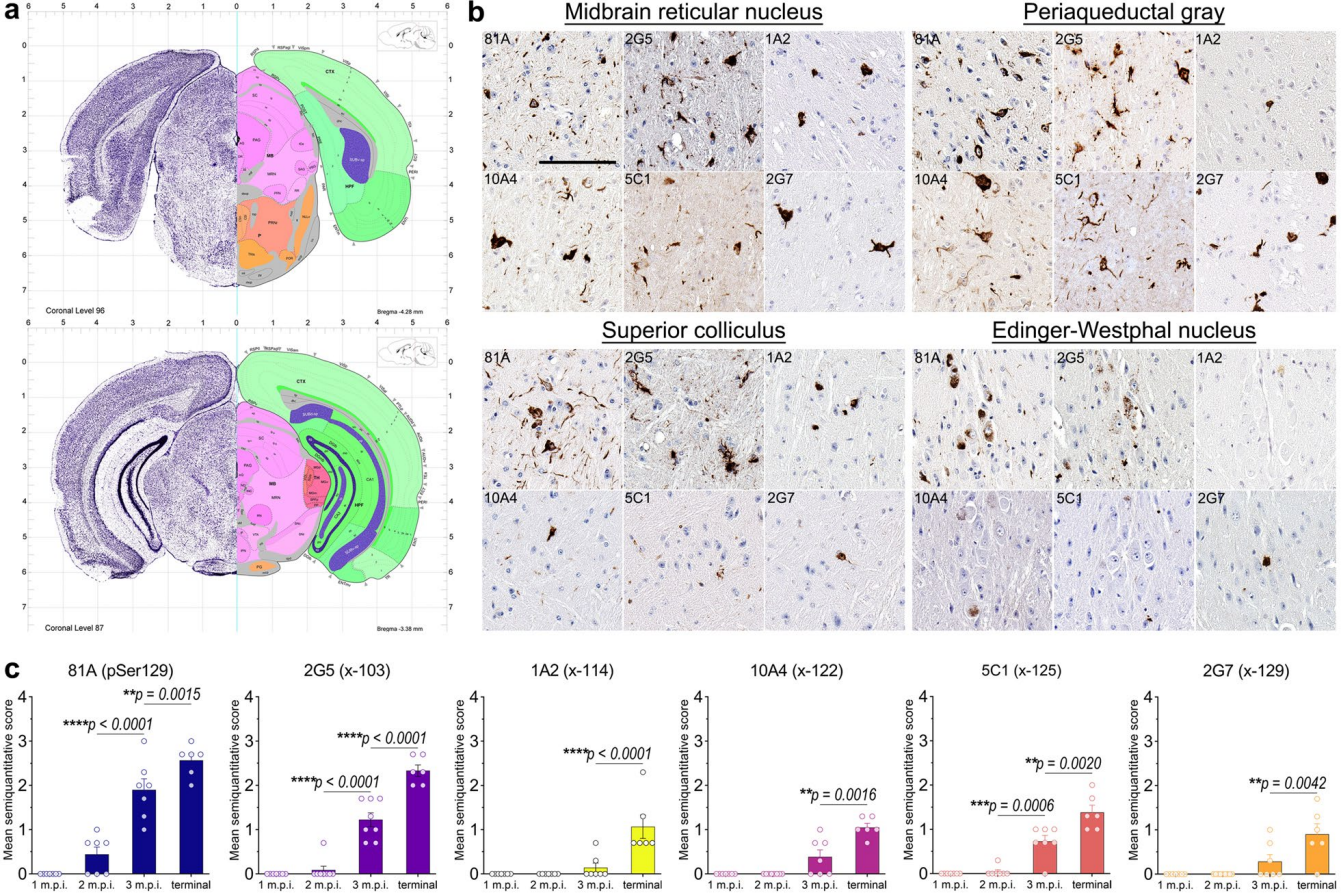
Accumulation of αSynΔC in the midbrain of end stage TgM83^+/−^ mice after peripheral intramuscular PFF induction. (**a**) Nissl (left) and anatomical annotations (right) of coronal section panels from the Allen reference atlas of the adult mouse brain, midbrain regions analyzed in study are annotated in pink. (**b**, **c**) Representative IHC images and semi-quantification of antibody immunoreactivity comparing the pathological αSyn deposition of TgM83^+/−^ mice at end stage. Antibodies specific for αSyn phosphorylated at Ser129 (81A) and αSynΔC truncated at 103 (2G5), 114 (1A2), 122 (10A4), 125 (5C1) and 129 (2G7) were used for IHC, as indicated. Sections were counterstained with hematoxylin. Scale bar = 100 μm. Data expressed as mean +/− SEM. Results were analyzed using 2-way ANOVA and corrected for multiple comparisons using Tukey’s test. n = 8 (4F:4M); 9 (5F:4M); 8 (4F:4M); 8 (5F:3M). *p < 0.05; **p < 0.01; ***p < 0.001; ****p < 0.0001

To determine whether αSynΔC positive inclusions occurred in more distal anatomically connected regions relation to the site of injection, tissue from the midbrain was probed, indicated in pink in the annotated reference in Fig. 4a, of IM PFF-injected TgM83^+/−^ mice with antibodies targeting pSer129 and αSynΔC-103, -114, -122, -125 and -129. At end stage, all forms of C-terminally truncated αSyn investigated in this study were detected in the midbrain (Fig. 4b–c). Analysis of the midbrain reticular nucleus (MRN), periaqueductal gray (PAG), superior colliculus (SCs) and Edinger Westphal nucleus (EW), regions densely populated with pSer129 positive pathology, revealed dramatic heterogeneity in the type of αSynΔC inclusions detected (Fig. 4b). The MRN was highest in 2G5 pathology and moderate in 1A2, 10A4, 5C1 and 2G7 pathology (Fig. 4b). The PAG was high in 2G5, 10A4, 5C1 and 2G7 pathology, and low in 1A2 pathology (Fig. 4b). The SCs was high in 2G5 pathology, moderate in 1A2 pathology, and low in 10A4, 5C1 and 2G7 pathology (Fig. 4b). The EW nucleus was high in 2G5 pathology and low in 1A2, 10A4, 5C1 and 2G7 pathology (Fig. 4b). Across the measured timepoints, pSer129 pathology in the pons exhibited a pattern of gradational increase that was congruent for αSynΔC-positive pathology (Fig. 4c).

### Humanized APOE/M83^+/−^ mice develop mature αSyn inclusion pathology after intramuscular inoculation with PFFs

To investigate the effect of human ApoE isoforms on αSynΔC positive pathology, we generated mouse models of synucleinopathy with the background of different human APOE genotypes, by backcrossing the offspring of M83^+/+^ mice and homozygous humanized APOE ε4 or ε3 mice with M83^+/−^ mice for at least 2 generations to APOE ε4^+/+^/M83^+/−^ and APOE ε3^+/+^/M83^+/−^ mice (see Materials and Methods). Since humanized APOE mice are on a BL6 background, M83^+/−^ on a BL6 background were also generated for comparison. At 2 months of age, these mice were unilaterally IM injected with human αSyn PFFs. The mice were aged until the onset of fatal motor symptoms, whereupon the brains and spine were collected for IHC analysis.

**Fig. 5:**
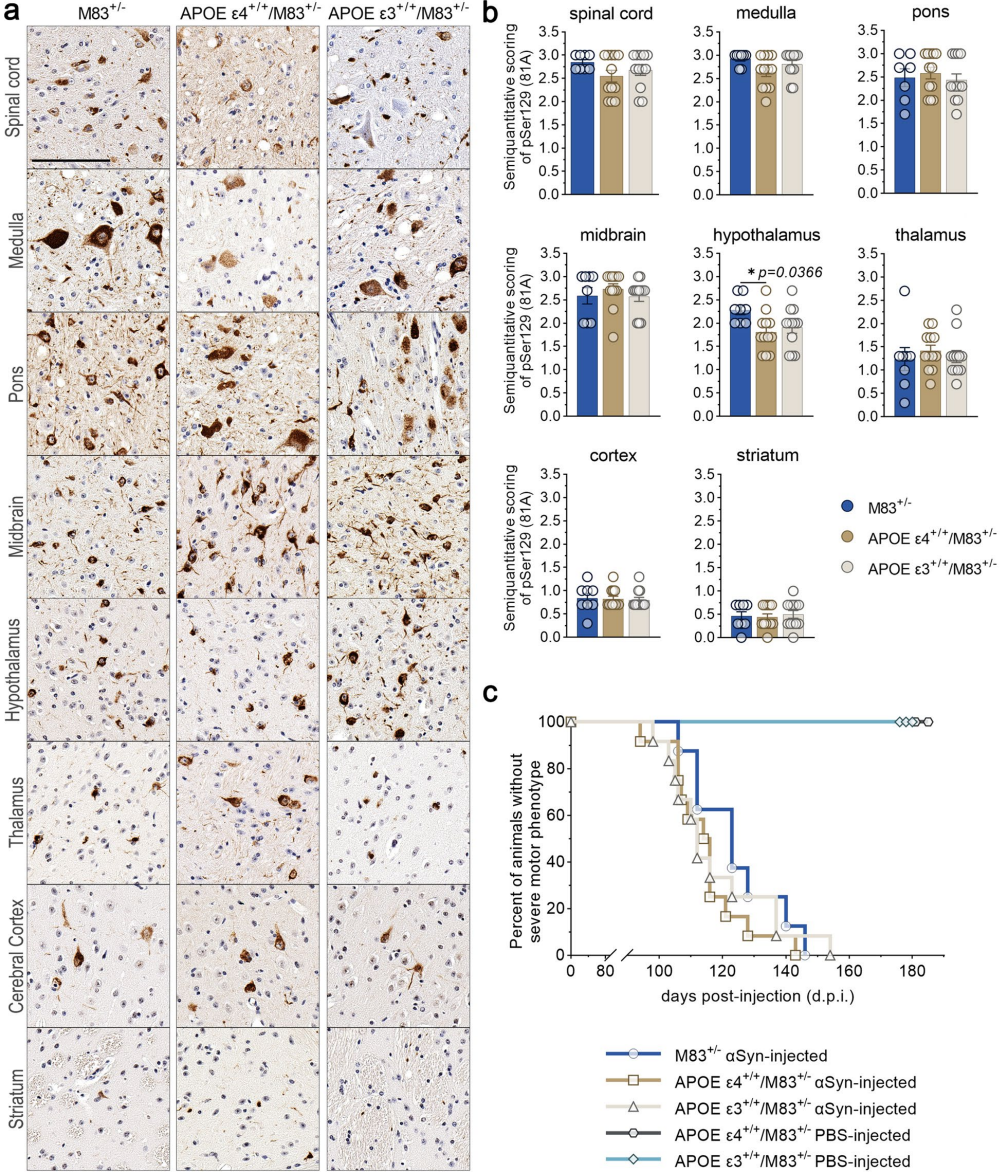
CNS accumulation of αSyn inclusion pathology in APOE ε4^+/+^/M83^+/−^ and APOE ε3^+/+^/M83^+/−^ mice after intramuscular inoculation with PFFs. (**a**) Representative images of immunostaining with an antibody specific for αSyn phosphorylated at serine 129 (81A). Brains and spinal cord from APOE ε3^+/+^/M83^+/−^ and APOE ε4^+/+^/M83^+/−^ mice were assessed for relative pathological burden in M83^+/−^mice. Scale bar = 100 μm. (**b**) Semi-quantification of 81A (pSer129) immunoreactivity in the spinal cord, medulla, pons, midbrain, hypothalamus, thalamus, cortex and striatum of PFF-injected M83^+/−^, APOE ε4^+/+^/M83^+/−^ and APOE ε3^+/+^/M83^+/−^ mice were scored by 3 independent observers. Results were analyzed using 1-way ANOVA and corrected for multiple comparisons using Tukey’s test. Data represents means +/− SEM*p < 0.05; **p < 0.01; ***p < 0.001; ****p < 0.0001. (**c**) Kaplan–Meier analysis of motor phenotype development in PFF-injected M83^+/−^ (n = 8; median survival, 123 days after injection), APOE ε3^+/−^/M83^+/−^ (n = 12; median survival, 112 days after injection), and APOE ε4^+/−^/M83^+/−^ mice (n = 12; median survival, 115 days after injection) see Table 2. Overall Log-rank (Mantel–Cox) p = 0.4904

To determine the profile of pathologic αSyn in these mice, brains and spinal cords from APOE ε3^+/+^/M83^+/−^ and APOE ε4^+/+^/M83^+/−^ mice injected with PFFs were assessed for markers of mature αSyn inclusion pathology using antibodies directed towards pSer129 (81A), as a measure of disease-associated αSyn, αSyn at the N-terminus (2H6) and C-terminus (3H19), as well as sequestrasome1 (p62). M83^+/−^, APOE ε4^+/+^/M83^+/−^ and APOE ε3^+/+^/M83^+/−^ mice showed robust and extensive, widespread αSyn pathology at end stage (Fig. 5a, Additional file 1: Fig. S2). APOE ε3^+/+^/M83^+/−^ and APOE ε4^+/+^/M83^+/−^ mice injected with PBS did not develop αSyn pathology (Additional file 1: Fig. S3). Semi-quantification of 81A pathology revealed a significantly lower level of hypothalamic inclusions in APOE ε4^+/+^/M83^+/−^ mice compared to M83^+/−^ mice (Fig. 5b), however, all other regions were relatively equal. Analysis of the onset of a motor phenotype revealed no difference between PFF-injected M83^+/−^ mice (n = 8; median survival, 123 days after injection), APOE ε3^+/+^/M83^+/−^ (n = 12; median survival, 112 days after injection), or APOE ε4^+/+^/M83^+/−^ mice (n = 12; median survival, 115 days after injection) (Fig. 5c).

### Regionally dynamic αSynΔC immunoreactive pathology is differentially regulated by APOE genotype

**Fig. 6:**
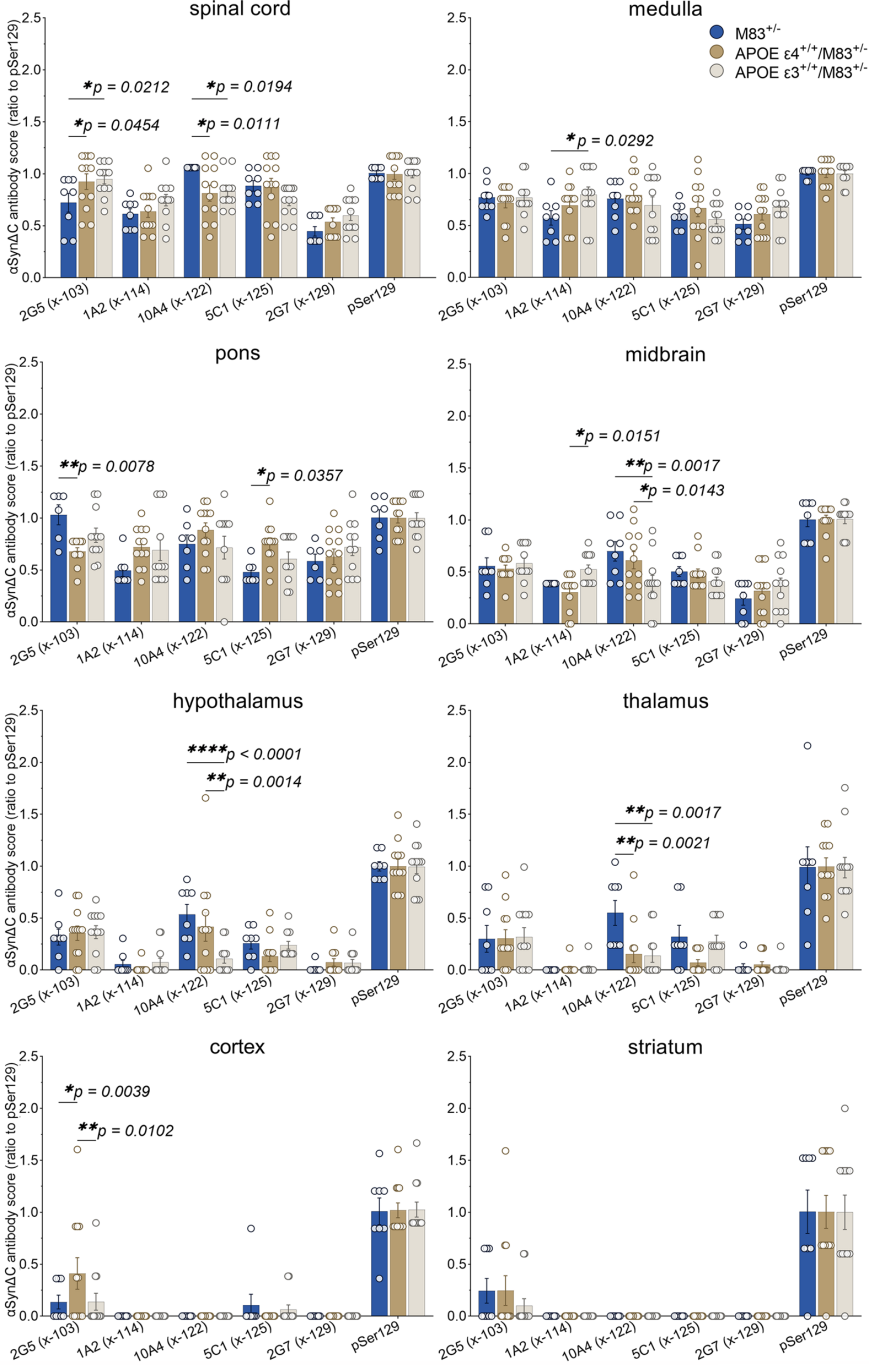
Regionally dynamic αSynΔC immunoreactive pathology is differentially regulated by APOE genotype. The burden of αSynΔC positive inclusions in the spinal cord, medulla, pons, midbrain, hypothalamus, thalamus, cortex and striatum of PFF-injected M83^+/−^, APOE ε4^+/+^/M83^+/−^ and APOE ε3^+/+^/M83^+/−^ mice were scored by 3 independent observers. Averaged scores were normalized to 81A (pSer129) and analyzed using 2-way ANOVA and corrected using Tukey’s multiple comparisons test. Data represents means +/− SEM. *p < 0.05; **p < 0.01; ***p < 0.001; ****p < 0.0001

**Fig. 7:**
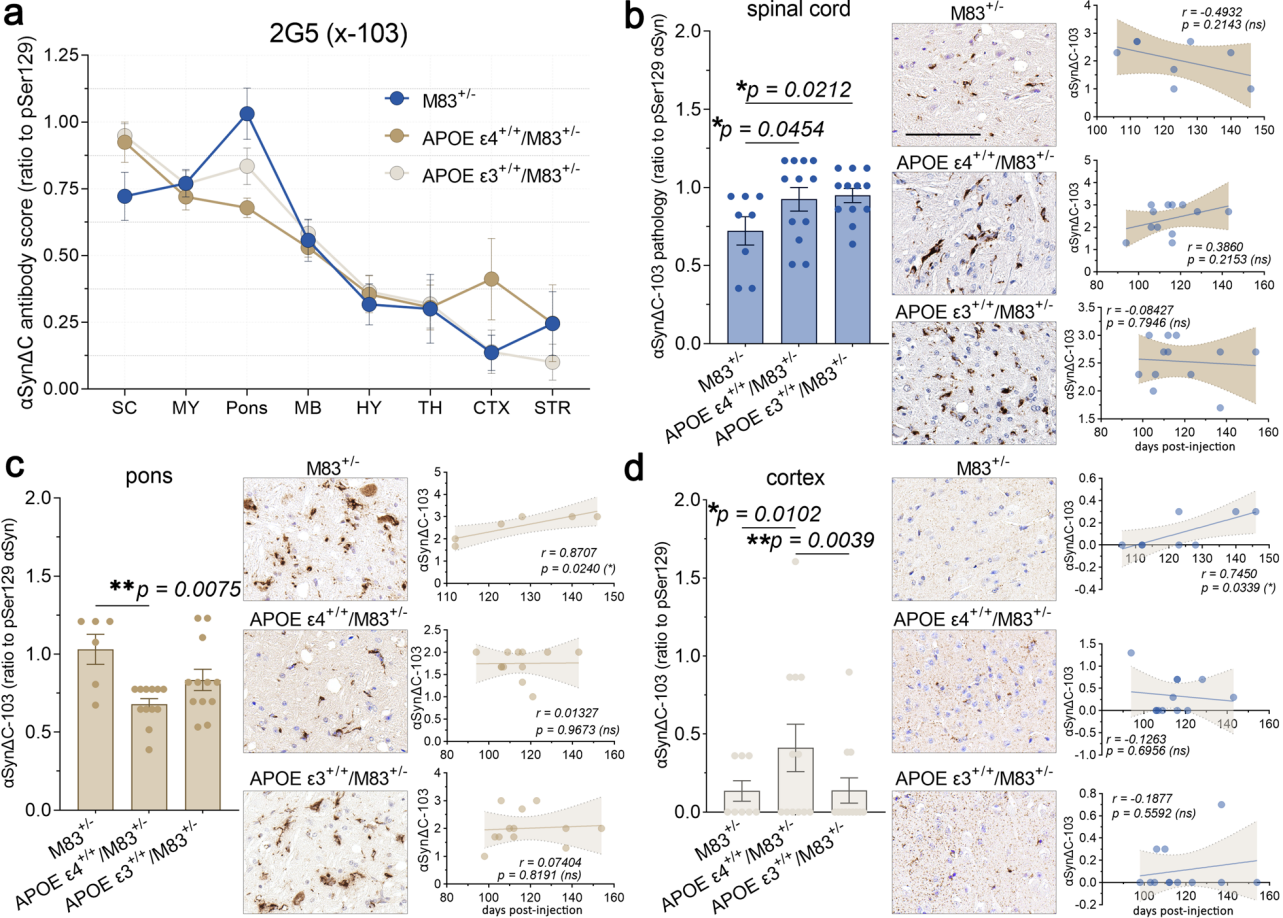
αSynΔC-103 immunoreactivity in the pons and cortex is associated with survival in time in M83^+/−^ intramuscular injected with PFFs, but not in APOE ε4^+/+^/M83^+/−^ or APOE ε3^+/+^/M83^+/−^. (**a**) Graph summarizing burden of αSynΔC-103 positive inclusions in the spinal cord (SC), medulla (MY), pons, midbrain (MB), hypothalamus (HY), thalamus (TH), cortex (CTX) and striatum (STR) of PFF-injected M83^+/−^, APOE ε4^+/+^/M83^+/−^ and APOE ε3^+/+^/M83^+/−^ mice depicted as mean with SEM. (**b**) Semi-quantification and representative images of αSynΔC-103 immunoreactivity with correlational analysis between spinal αSynΔC-103 immunoreactivity and d.p.i. for each cohort. (**c**) Semi-quantification and representative images of αSynΔC-103 immunoreactivity with correlational analysis between αSynΔC-103 immunoreactivity in the pons and d.p.i. for each cohort. (**d**) Semi-quantification and representative images of αSynΔC-103 immunoreactivity with correlational analysis between αSynΔC-103 immunoreactivity in the cortex and d.p.i. for each cohort. Results were analyzed using 2-way ANOVA and corrected using Tukey’s multiple comparisons test; data represent means +/− SEM. Pearson correlation coefficients between αSynΔC positivity and d.p.i. were computed with the assumption that data are sampled from Gaussian distribution. Pearson r and two-tailed p-values are indicated on graph. Simple linear regression is shown with dotted lines depicting 95% confidence intervals. n.s. not significant; *p < 0.05; **p < 0.01; ***p < 0.001; ****p < 0.0001. Scale bar = 100 μm

In order to compare the overall burden of αSynΔC positive inclusions, IHC analysis was performed using antibodies targeted towards αSyn C-terminally truncated at residue 103, 114, 122, 125, and 129. Regions within the spine and brain, ranging in distance to the injection site including the spinal cord, medulla, pons, midbrain, hypothalamus, thalamus, cortex and striatum were analyzed. Immunostained sections were evaluated via semi-quantitative analysis by 3 independent observers and averaged score were normalized to 81A (Fig. 6). The data for all the αSynΔC antibodies, regions analyzed and genotypes are shown in Fig. 6 for easier direct comparison. Our data revealed that αSynΔC immunoreactivity was regionally dynamic, and that this was altered based on APOE genotype. This was analyzed in greater detail for each αSynΔC antibody. αSynΔC-103 (2G5) immunoreactivity was higher in the spine, hindbrain and midbrain for all cohorts compared to forebrain regions (Fig. 7a). For regions that displayed genotype-specific differences such as the spinal cord, pons and cortex, we performed additional statistical analyses (Fig. 7b–c). In the spine, both APOE ε3^+/+^/M83^+/−^ and APOE ε4^+/+^/M83^+/−^ mice exhibited significantly higher levels of 2G5 pathology compared to M83^+/−^ mice, however, αSynΔC-103 pathology in this region failed to show a significant correlation to days to terminal motor symptoms (Fig. 7b). In the pons, αSynΔC-103 immunoreactivity was higher in M83^+/−^ than in APOE ε4^+/+^/M83^+/−^ mice (Fig. 7c), while, cortical αSynΔC-103 positive pathology was highest in APOE ε4^+/+^/M83^+/−^ mice (Fig. 7d). Interestingly, αSynΔC-103 immunoreactivity in the pons and cortex was associated with elongated survival in M83^+/−^, but not in APOE ε3^+/+^/M83^+/−^ or APOE ε4^+/+^/M83^+/−^ (Fig. 7c–d).

**Fig. 8:**
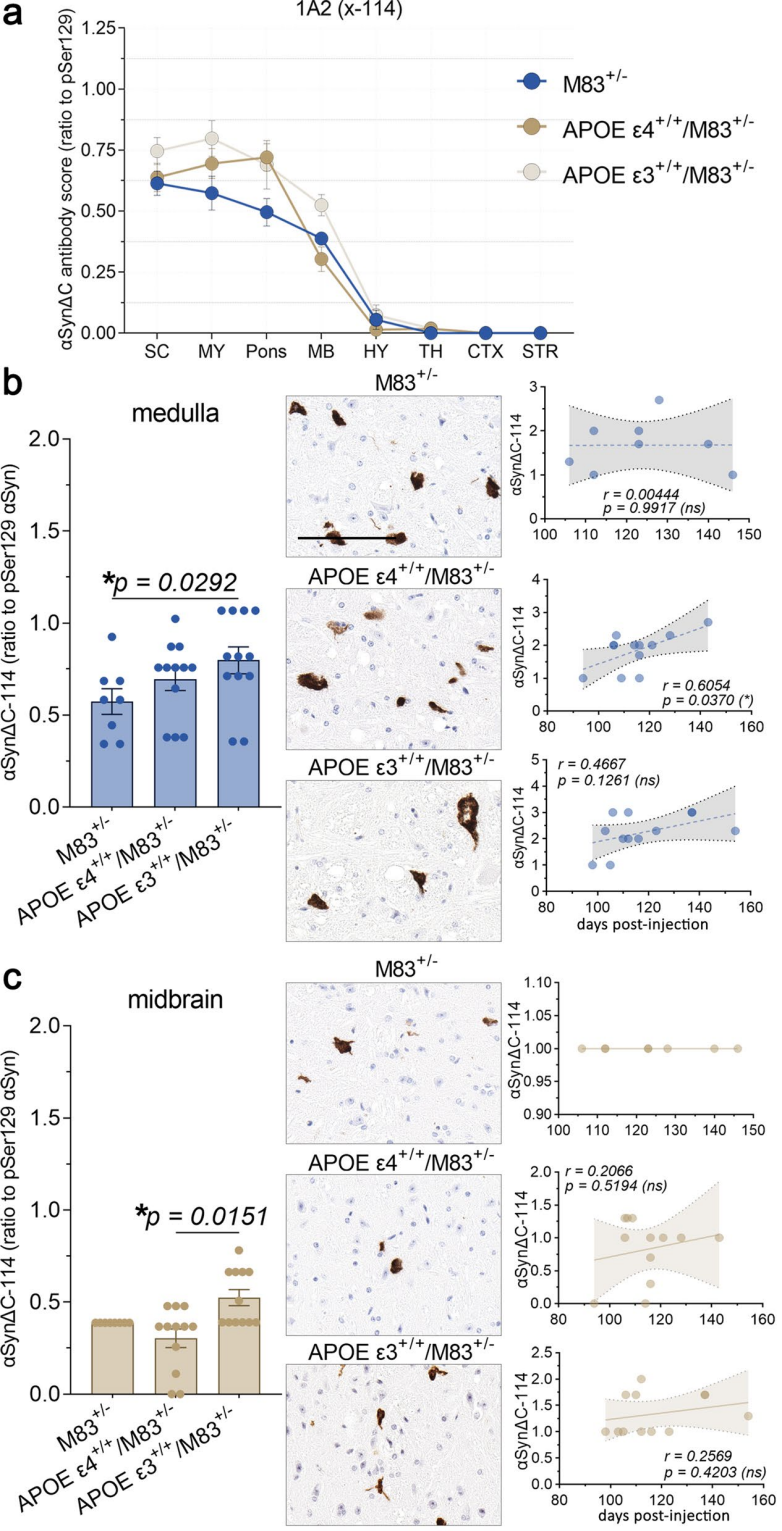
Medullary αSynΔC-114 inclusion pathology is positively correlated with onset of motor symptoms in APOE ε4^+/+^/M83^+/−^ mice. (**a**) Graph summarizing burden of αSynΔC-114 positive inclusions in the spinal cord (SC), medulla (MY), pons, midbrain (MB), hypothalamus (HY), thalamus (TH), cortex (CTX) and striatum (STR) of PFF-injected M83^+/−^, APOE ε4^+/+^/M83^+/−^ and APOE ε3^+/+^/M83^+/−^ mice depicted as mean with SEM. (**b**) Semi-quantification and representative images of αSynΔC-114 immunoreactivity in the medulla with correlational analysis between αSynΔC-114 immunoreactivity and d.p.i. for each cohort. (**c**) Semi-quantification and representative images of αSynΔC-114 immunoreactivity with correlational analysis between αSynΔC-114 immunoreactivity in the midbrain and d.p.i. for each cohort. M83^+/−^ horizontal line. Results were analyzed using 2-way ANOVA and corrected using Tukey’s multiple comparisons test; data represent means +/− SEM. Pearson correlation coefficients between αSynΔC positivity and d.p.i. were computed with the assumption that data are sampled from Gaussian distribution. Pearson r and two-tailed *p*-values are indicated on graph. Simple linear regression is shown with dotted lines depicting 95% confidence intervals. n.s. not significant; *p < 0.05; **p < 0.01; ***p < 0.001; ****p < 0.0001. Scale bar = 100 μm

Evaluation of αSynΔC-114 (1A2) immunostaining revealed moderate positivity in the spine, hindbrain and midbrain and relative few positive inclusions within the forebrain for all cohorts (Fig. 8a). For regions that displayed genotype-specific differences such as the medulla and midbrain, we performed additional statistical analyses (Fig. 8b, c). APOE ε3^+/+^/M83^+/−^ mice had greater 1A2-positive inclusion pathology in the medulla compared to M83^+/−^ (Fig. 8b) and in the midbrain compared to APOE ε4^+/+^/M83^+/−^ mice (Fig. 8c). αSynΔC-114 immunoreactivity in the medulla but not in the midbrain was positively associated with days to terminal motor symptoms in APOE ε4^+/+^/M83^+/−^ mice (Fig. 8b, c). There was no association between 1A2-positive inclusion pathology and days to terminal motor symptoms post PFF injection for the other genotypes.

**Fig. 9:**
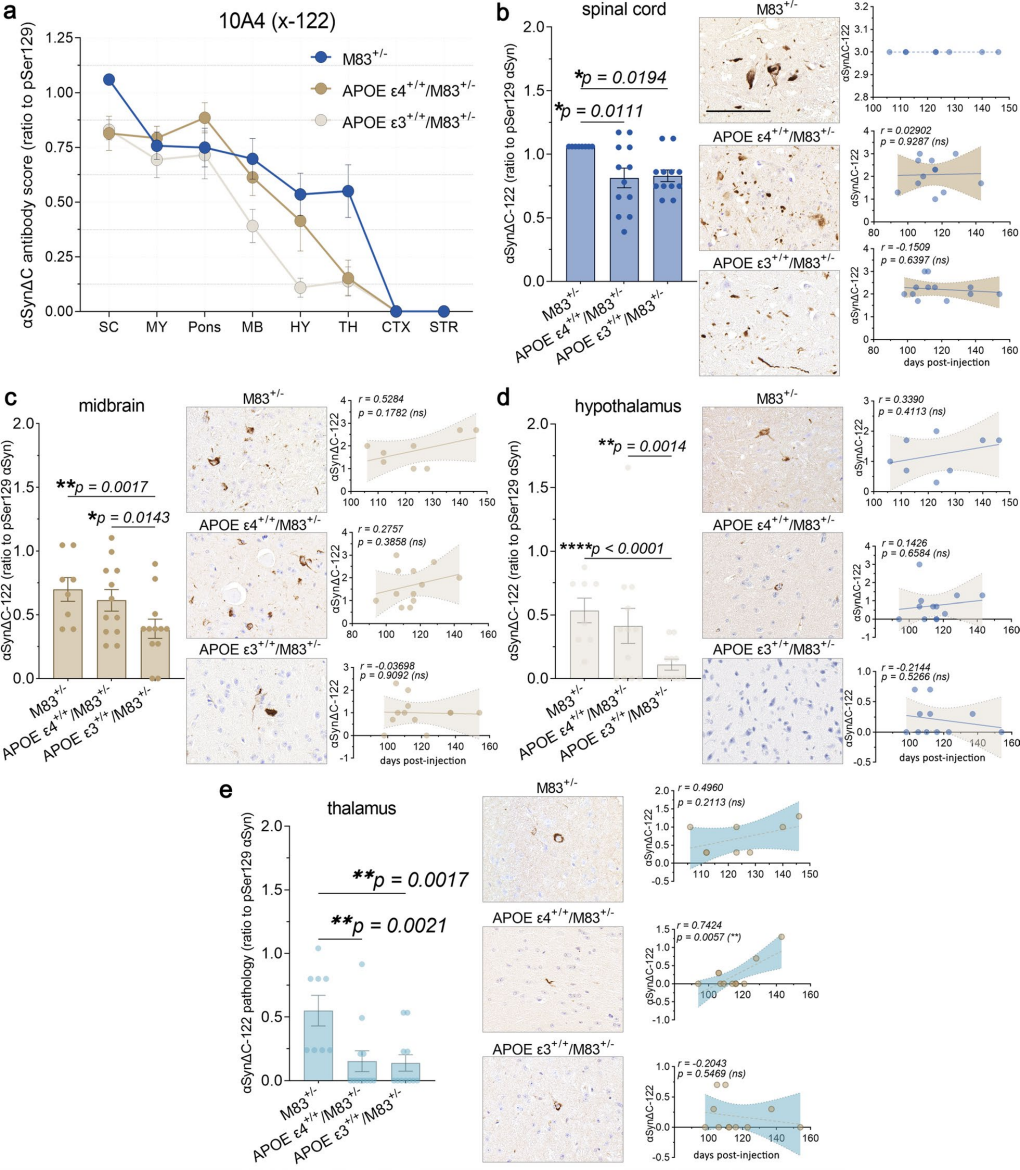
αSynΔC-122 immunoreactivity is constrained to the spine and brainstem and is significantly lower in APOE ε3^+/+^/M83^+/−^ mice compared to M83^+/−^. (**a**) Graph summarizing burden of αSynΔC-122 positive inclusions in the spinal cord (SC), medulla (MY), pons, midbrain (MB), hypothalamus (HY), thalamus (TH), cortex (CTX) and striatum (STR) of PFF-injected M83^+/−^, APOE ε4^+/+^/M83^+/−^ and APOE ε3^+/+^/M83^+/−^ mice depicted as mean with SEM. (**b**) Semi-quantification and representative images of αSynΔC-122 immunoreactivity with correlational analysis between spinal αSynΔC-122 immunoreactivity and d.p.i. for each cohort. (**c**) Semi-quantification and representative images of αSynΔC-122 immunoreactivity with correlational analysis between αSynΔC-122 immunoreactivity in the midbrain and d.p.i. for each cohort. (**d**) Semi-quantification and representative images of αSynΔC-122 immunoreactivity with correlational analysis between αSynΔC-122 immunoreactivity in the hypothalamus and d.p.i. for each cohort. (**e**) Semi-quantification and representative images of αSynΔC-122 immunoreactivity with correlational analysis between αSynΔC-122 immunoreactivity in the thalamus and d.p.i. for each cohort. Results were analyzed using 2-way ANOVA and corrected using Tukey’s multiple comparisons test; data represent means +/− SEM. Pearson correlation coefficients between αSynΔC positivity and d.p.i. were computed with the assumption that data are sampled from Gaussian distribution. Pearson r and two-tailed p-values are indicated on graph. Simple linear regression is shown with dotted lines depicting 95% confidence intervals. n.s. not significant; *p < 0.05; **p < 0.01; ***p < 0.001; ****p < 0.0001. Scale bar = 100 μm

Examination of αSynΔC-122 (10A4) immunostaining revealed a higher burden of positive pathology in the spine, hindbrain and midbrain for all cohorts compared to forebrain regions (Fig. 9a). For regions that displayed genotype-specific differences, such as the spinal cord, midbrain, hypothalamus and thalamus, we performed additional statistical analyses (Fig. 9b–e). M83^+/−^ mice demonstrated a greater burden of 10A4 positive inclusion pathology compared to APOE ε3^+/+^/M83^+/−^ and APOE ε4^+/+^/M83^+/−^ mice in the spine (Fig. 9b). In the midbrain and hypothalamus, M83^+/−^ and APOE ε4^+/+^/M83^+/−^ mice both exhibited greater levels of αSynΔC-122 (10A4) positive immunostaining compared to APOE ε3^+/+^/M83^+/−^ mice (Fig. 9c–d). However, in the thalamus, M83^+/−^ mice again demonstrated a greater burden of 10A4 positive inclusion pathology in comparison to APOE ε3^+/+^/M83^+/−^ and APOE ε4^+/+^/M83^+/−^ mice (Fig. 9e). Interestingly, thalamic 10A4 positivity in APOE ε4^+/+^/M83^+/−^ mice positively correlated with a later onset of motor symptoms.

**Fig. 10:**
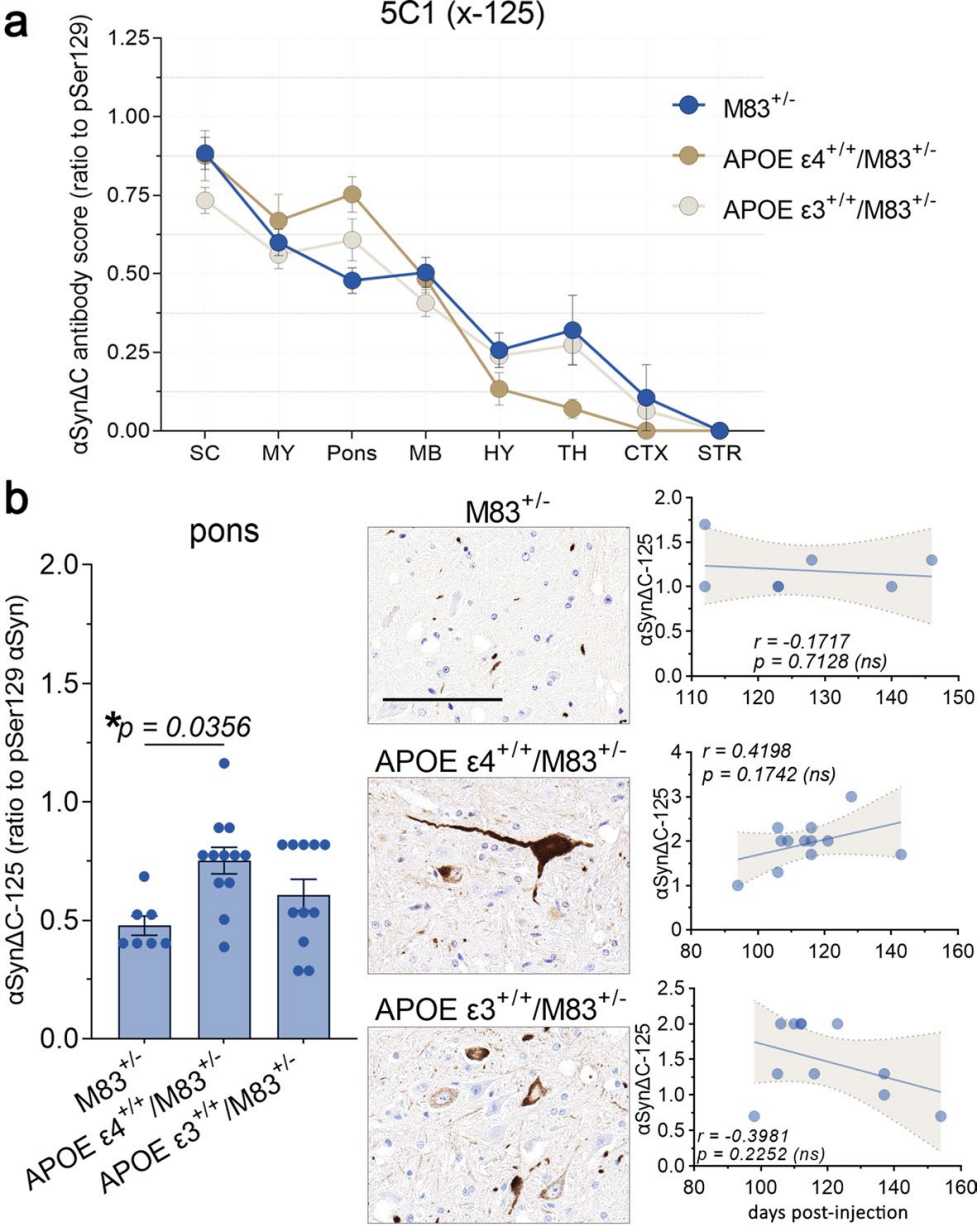
APOE ε4^+/+^/M83^+/−^ mice develop a greater burden of αSynΔC-125 positive pontine pathology compared to M83^+/−^mice. (**a**) Graph summarizing burden of αSynΔC-125 positive inclusions in the spinal cord (SC), medulla (MY), pons, midbrain (MB), hypothalamus (HY), thalamus (TH), cortex (CTX) and striatum (STR) of intramuscular PFF-injected M83^+/−^, APOE ε4^+/+^/M83^+/−^ and APOE ε3^+/+^/M83^+/−^ mice depicted as mean with SEM. (**b**) Semi-quantification and representative images of αSynΔC-125 immunoreactivity with correlational analysis between αSynΔC-125 immunoreactivity in the pons and d.p.i. for each cohort. Results were analyzed using 2-way ANOVA and corrected using Tukey’s multiple comparisons test; data represent means  +/-SEM. Pearson correlation coefficients between αSynΔC positivity and d.p.i. were computed with the assumption that data are sampled from Gaussian distribution. Pearson r and two-tailed p-values are indicated on graph. Simple linear regression is shown with dotted lines depicting 95% confidence intervals. n.s. not significant; *p < 0.05; **p < 0.01; ***p < 0.001; ****p < 0.0001. Scale bar = 100 μm

Analysis of αSynΔC-125 (5C1) immunostaining revealed a decreasing trend of positive pathology in all cohorts, as distance from injection site increased (Fig. 10a). Further analysis revealed that APOE ε4^+/+^/M83^+/−^ mice exhibited greater pontine 5C1-positive pathology compared to M83^+/−^ mice, however, there was no correlation with onset of terminal motor symptoms post PFF injection (Fig. 10b).αSynΔC-129 (2G7) immunostaining of αSyn pathological inclusions was also more abundant closer to the injection site, including the spine, hindbrain and midbrain compared to forebrain regions (Fig. 6) but there was no statistically significant difference between any of the genotypes injected with PFFs.

## Discussion

**Fig. 11:**
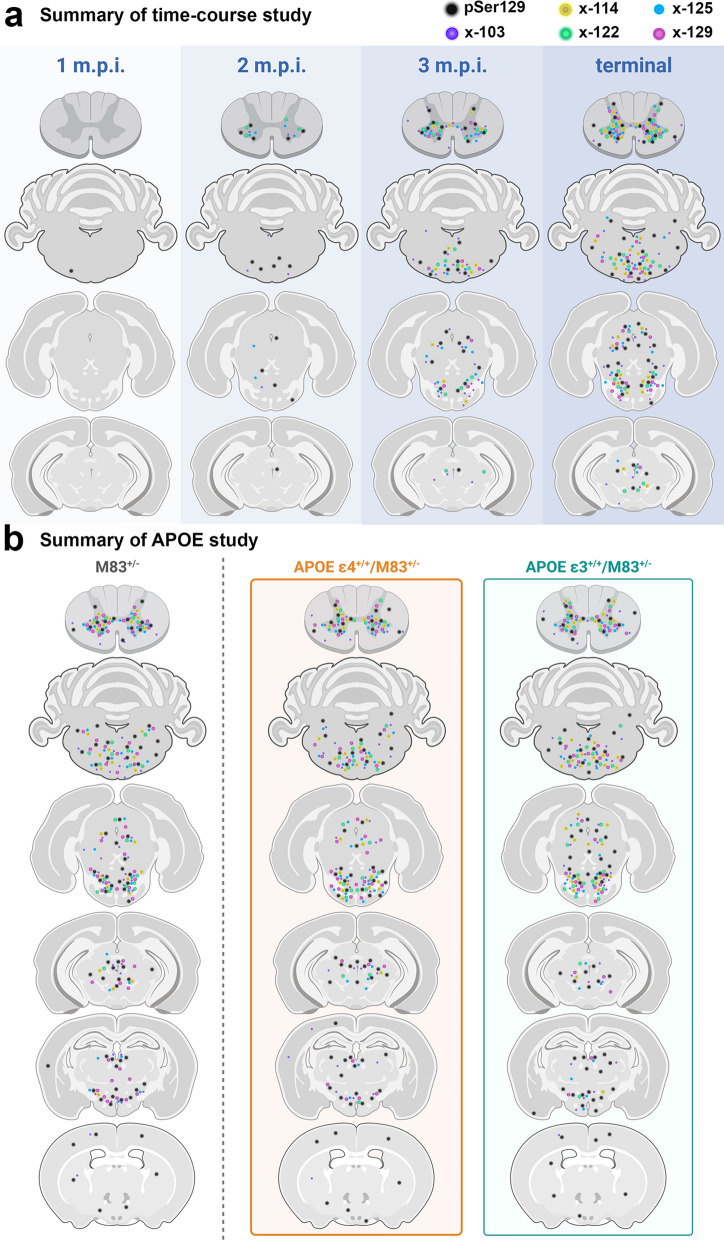
Schematic summary of the observed pathological findings in various CNS regions, using C-terminally truncated αSyn-specific antibodies. Diagram illustrating data from the (**a**) time-course study and (**b**) the APOE study, depicting the relative levels of detected C-terminally truncated αSyn across the neuroaxis. Created with Biorender.com

Our study investigated the spatiotemporal characterization of a series of αSynΔC species that occurs during pathogenesis in a peripheral to CNS seeded model of synucleinopathies. This IM seeding model provides the ability to assess changes associated with the processive spatial formation of αSyn pathology along predicted neuroanatomical pathways following the prion-type neuroinvasion of the CNS. Our findings revealed concurrent accumulation of αSynΔC with αSyn phosphorylated at pSer129, as well as an APOE isoform-dependent alteration of αSynΔC-positive inclusion accumulation (Fig. 11), suggesting that proteolytic processing of αSyn may be a key component to the early stages of pathology and may differentiate stages of disease progression. Our findings show that αSynΔC-103, -122 and -125 occur at detectable levels in αSyn aggregrates as early as 2 m.p.i. when the formation of αSyn pathology starts, and that by the terminal endpoint, αSynΔC-103, -114, -122, -125 and -129 are all observed, at various levels, in pathological inclusions in synaptically connected regions, throughout the neuroaxis (Fig. 11a). Furthermore, we demonstrate that while αSyn-APOE mice develop mature αSyn inclusion pathology and paralysis, at a rate not significantly different from TgM83^+/−^ controls, the regionality and disposition of αSynΔC positive pathology is indeed influenced by the presence of different APOE alleles (Fig. 11b), although the mechanism is unclear. This suggests a role for ApoE isoforms in the regulation of αSyn metabolism; further studies are needed to elaborate upon this relationship.

In this study, we characterized the temporal and spatial accumulation of carboxy-truncated αSyn at specific residues (103, 114, 122, 125, and 129) in the CNS after PFF injection into the hindleg gastrocnemius muscle of different TgM83 ^+/−^ based mouse models. Intramuscular PFF injection is a useful tool for modeling the prion-like spread of seeds through connected neuron populations, as this method of neuroinvasion follows defined groups of neurons in the spinal cord, thereby not confounded by possible dissemination that may occur with intracerebral injections. For spatiotemporal analysis in our time-course study, we analyzed the mice at 1-, 2-, and 3- months post injection, as well as at terminal stage (approximately 4 months), in regions increasingly distal from injection site. Previously, we have shown the temporal progression of αSyn inclusion pathology in the CNS after hindleg IM injection of PFFs in TgM83^+/−^ mice, with subsequent astroglial and microglial activation [45]. We demonstrated that peripheral PFF injection induced pSer129 + αSyn inclusion pathology in the spine and midbrain by 2 m.p.i., and the ventral thalamus and cortex by 3 m.p.i. Our findings have revealed that some forms of αSynΔC appear at timepoints concurrent with the onset of pSer129 accumulation, while others are not detected until later timepoints (Fig. 11). We have also shown that αSynΔC species progressively accumulate, predominantly in neuroanatomically connected regions, and, by end stage, many reach regions distal from the injection site (Fig. 11). Possible molecular explanations for this include a prion-like mechanism of intercellular spread that is recapitulated in the templated accumulation of αSynΔC, wherein truncated species are recruited into fibril assembly. In this model, proteolytic processing of αSyn occurs prior to fibril formation, meaning that aggregate elongation would be dictated by enzymatic activity. Alternatively, proteolytic degradation of αSyn may occur after fibril formation, where aggregated, full length αSyn proteins are partially degraded by proteases, which remove segments from the C-terminus, exposing the epitopes detected by αSynΔC-specific antibodies.

Interestingly, C-truncated αSyn pathology presentation skyrockets in the spine by 3 m.p.i., especially αSyn truncated at residue 103, which is also when we first detected elevated levels of GFAP, cd11b, and Iba1 positivity [45]. These findings suggest that the accumulation of specific αSyn carboxy-truncated species, particularly αSynΔC-103, may play a critical role in the rapid stages of prion-like spread of αSyn pathology.  Furthermore, the temporal and spatial accumulation patterns of αSynΔC-103 positive inclusions differ from those of other αSynΔC positive inclusions examined in this study, suggesting that proteolysis of αSyn may occur in a specific and regulated manner during the progression of synucleinopathies. Moreover, the fact that the increase in detection of C-terminally truncated αSyn is temporally associated with the occurrence of neuroinflammation indicates that proteolysis may be a contributing factor to the pathogenesis of synucleinopathies by promoting a pro-inflammatory environment. These insights may have important implications for understanding the molecular mechanisms underlying synucleinopathies and developing targeted therapies aimed at preventing or slowing their progression. Future studies will investigate the role of the individual αSynΔC species in modulating the initiation and progression of prion-type αSyn pathogenesis and the impact on associated neuroinflammation.

## Supplementary Information

**Additional file 1**. **Figure S1**: Low magnification images depicting accumulation of C-terminally truncated αSyn in the spine of TgM83^+/−^ mice following intramuscular administration of PFFs. Representative IHC images comparing the pathological deposition of αSyn TgM83^+/−^ mice at 1-, 2-, 3- months post injection or terminal stage. Antibodies specific for αSyn phosphorylated at Ser129 (81A) and αSyn truncated at residues 103 (2G5), 114 (1A2), 122 (10A4), 125 (5C1) or 129 (2G7) were used for IHC, as indicated. Sections were counterstained with hematoxylin. Scale bar = 300 μm. **Figure S2**: Representative images of immunostaining with antibodies specific for αSyn (2H6 and 3H19) and p62/sequestrasome-1. Brains and spinal cord from APOE ε3^+/+^/M83^+/−^ and APOE ε4^+/+^/M83^+/−^ mice at end stage following intramuscular injection with PFFs were assessed for markers of mature αSyn inclusion pathology using antibodies directed towards αSyn at the N-terminus (2H6) and C-terminus (3H19), as well as sequestrasome-1 (p62). Sections were counterstained with hematoxylin. Scale bar = 100 μm. **Figure S3**: αSyn inclusion pathology was not detected in the CNS of PBS-injected APOE ε3^+/+^/M83^+/−^ and APOE ε4^+/+^/M83^+/−^ mice. Representative images of immunostaining from the spine and medulla of PBS-injected (a) APOE ε4^+/+^/M83^+/−^ and (b) APOE ε3^+/+^/M83^+/−^ mice. Tissue was analyzed with antibodies directed towards αSyn phosphorylated at Ser129 (81A), at the N-terminus (2H6) and C-terminus (3H19), as well as sequestrasome-1 (p62). Sections were counterstained with hematoxylin. Scale bar = 300 μm.

## Data Availability

The datasets used and analyzed during the current study are available from the corresponding author upon reasonable request.
