# Supplementary figures and images for: Carboxyl truncation of α-synuclein occurs early and is influenced by human APOE genotype in transgenic mouse models of α-synuclein pathogenesis
Source: Acta Neuropathol Commun. 2023 Jul 23;11:119. doi: 10.1186/s40478-023-01623-9 (PMC10363304; doi:10.1186/s40478-023-01623-9)

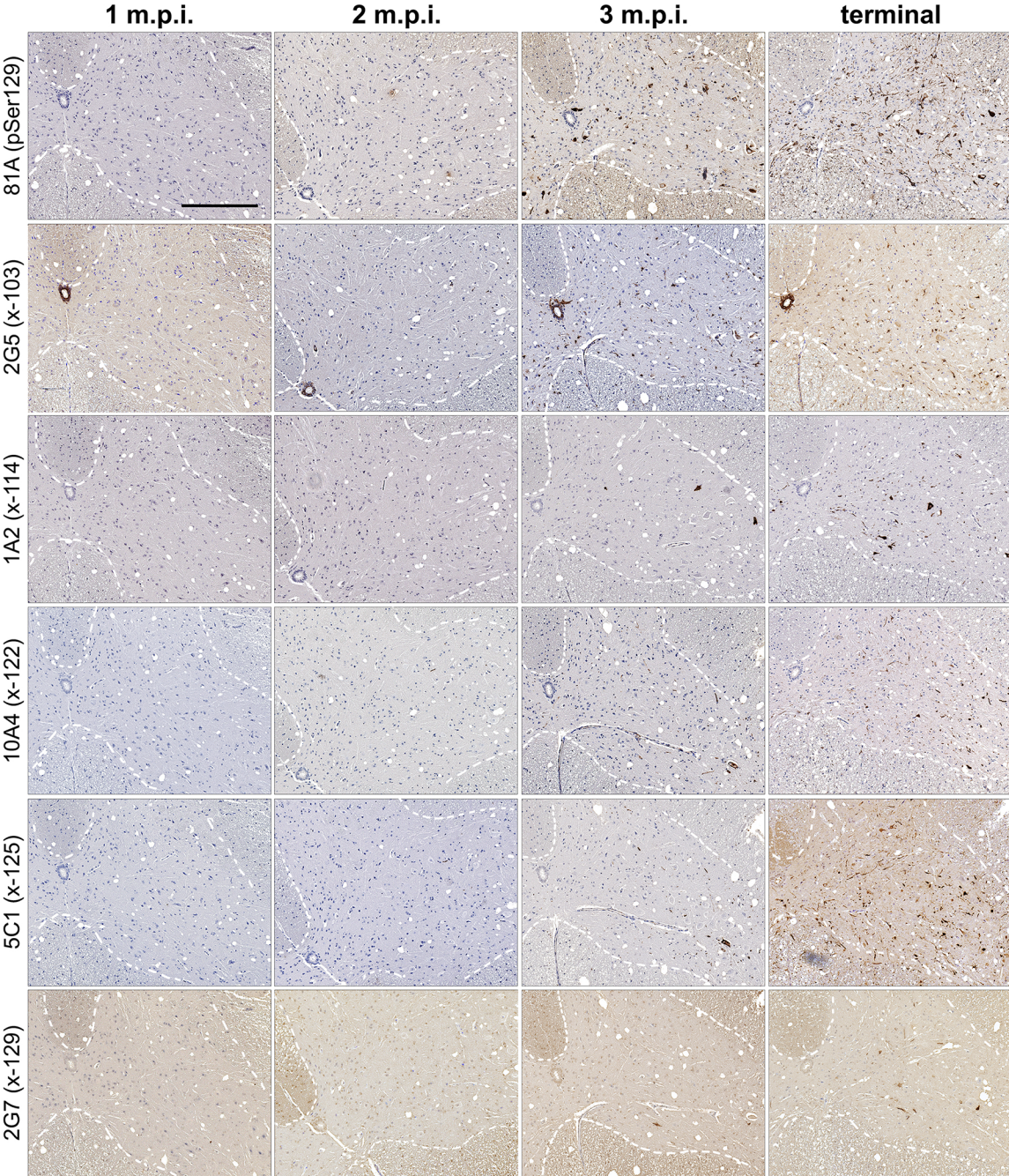

**Supplemental Figure 1**

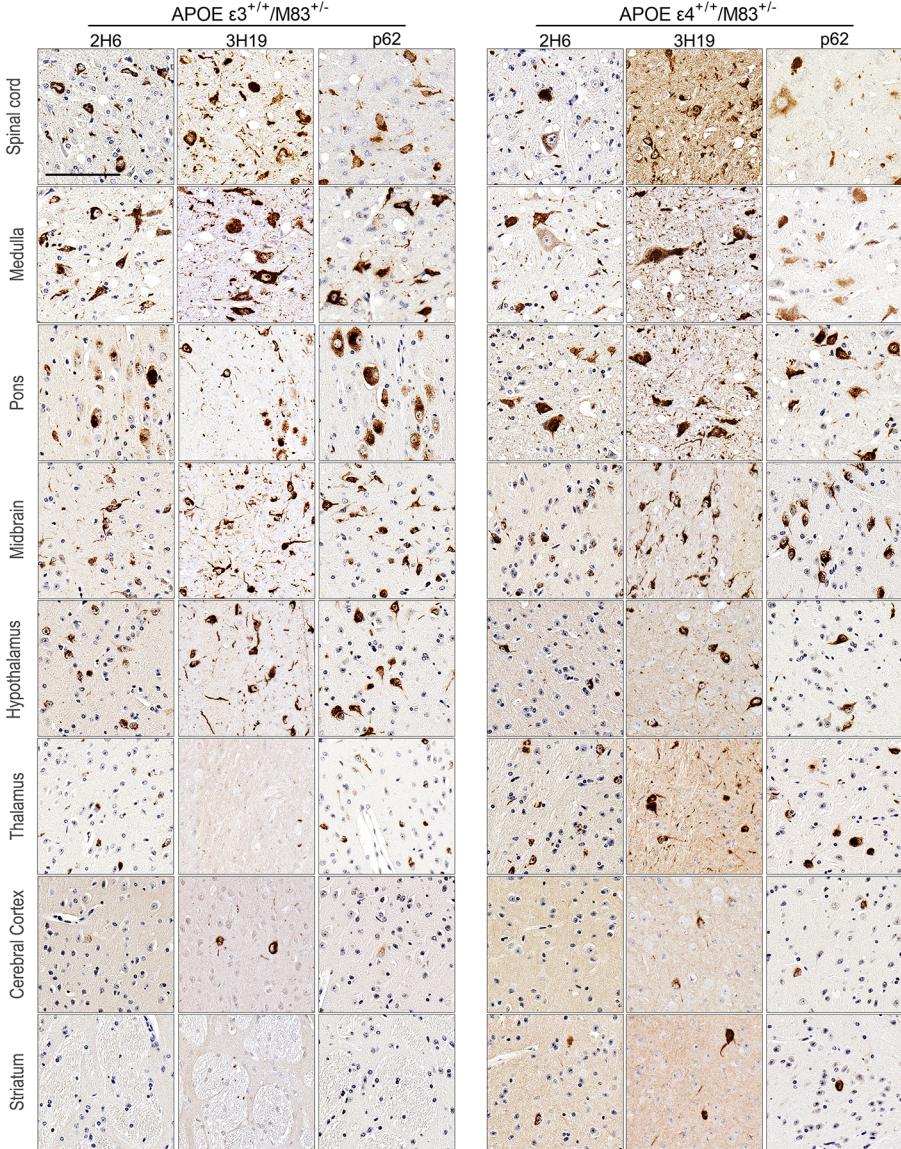

**Supplemental Figure 2**

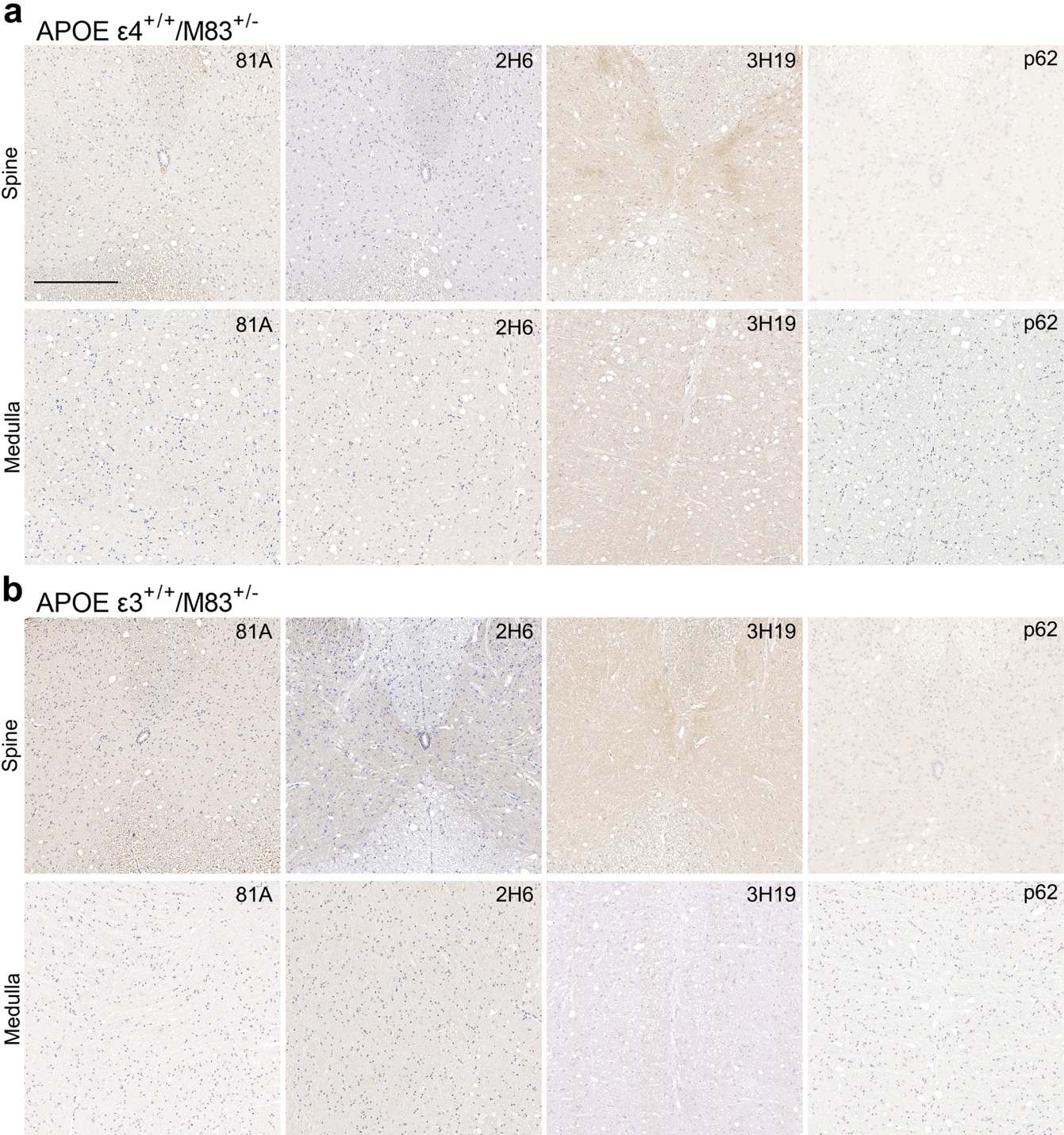

**Supplemental Figure 3**

Supplement: Supplementary file 1 — Additional file 1. Figure S1: Low magnification images depicting accumulation of C-terminally truncated αSyn in the spine of TgM83+/− mice following intramuscular administration of PFFs. Representative IHC images comparing the pathological deposition of αSyn TgM83+/− mice at 1-, 2-, 3- months post injection or terminal stage. Antibodies specific for αSyn phosphorylated at Ser129 (81A) and αSyn truncated at residues 103 (2G5), 114 (1A2), 122 (10A4), 125 (5C1) or 129 (2G7) were used for IHC, as indicated. Sections were counterstained with hematoxylin. Scale bar = 300 μm. Figure S2: Representative images of immunostaining with antibodies specific for αSyn (2H6 and 3H19) and p62/sequestrasome-1. Brains and spinal cord from APOE ε3+/+/M83+/− and APOE ε4+/+/M83+/− mice at end stage following intramuscular injection with PFFs were assessed for markers of mature αSyn inclusion pathology using antibodies directed towards αSyn at the N-terminus (2H6) and C-terminus (3H19), as well as sequestrasome-1 (p62). Sections were counterstained with hematoxylin. Scale bar = 100 μm. Figure S3: αSyn inclusion pathology was not detected in the CNS of PBS-injected APOE ε3+/+/M83+/− and APOE ε4+/+/M83+/− mice. Representative images of immunostaining from the spine and medulla of PBS-injected (a) APOE ε4+/+/M83+/− and (b) APOE ε3+/+/M83+/− mice. Tissue was analyzed with antibodies directed towards αSyn phosphorylated at Ser129 (81A), at the N-terminus (2H6) and C-terminus (3H19), as well as sequestrasome-1 (p62). Sections were counterstained with hematoxylin. Scale bar = 300 μm. [file 40478_2023_1623_MOESM1_ESM.pdf]
